# Systematic Review and Meta-Analyses of Incidence for Group B *Streptococcus* Disease in Infants and Antimicrobial Resistance, China

**DOI:** 10.3201/eid2611.181414

**Published:** 2020-11

**Authors:** Yijun Ding, Yajuan Wang, Yingfen Hsia, Neal Russell, Paul T. Heath

**Affiliations:** National Center for Children’s Health, Beijing, China (Y. Ding, Y. Wang);; University of London, London, UK (Y. Hsia, N. Russell, P.T. Heath);; Queen’s University Belfast, Belfast, Northern Ireland, UK (Y. Hsia)

**Keywords:** infants, newborns, respiratory infections, group B Streptococcus, GBS, bacteria, group B Streptococcus disease, streptococci, systematic review, meta-analyses, case-fatality rate, antimicrobial resistance, China

## Abstract

We performed a systematic review and meta-analysis of the incidence, case-fatality rate (CFR), isolate antimicrobial resistance patterns, and serotype and sequence type distributions for invasive group B *Streptococcus* (GBS) disease in infants <1–89 days of age in China. We searched the PubMed/Medline, Embase, Wanfang, and China National Knowledge Infrastructure databases for research published during January 1, 2000–March 16, 2018, and identified 64 studies. Quality of included studies was assessed by using Cochrane tools. Incidence and CFR were estimated by using random-effects meta-analyses. Overall incidence was 0.55 (95% CI 0.35–0.74) cases/1,000 live births, and the CFR was 5% (95% CI 3%–6%). Incidence of GBS in young infants in China was higher than the estimated global incidence (0.49 cases/1,000 live births) and higher than previous estimates for Asia (0.3 cases/1,000 live births). Our findings suggest that implementation of additional GBS prevention efforts in China, including maternal vaccination, could be beneficial.

Group B *Streptococcus* (GBS; *Streptococcus agalactiae*) is a major cause of illness and death in young infants worldwide (*1**–**3*). A recent systematic review reported the global incidence to be 0.49 cases/1,000 live births (*4*). It is estimated that this incidence results in »90,000 deaths (uncertainty death range 36,000–169,000) in infants every year (*5*). Furthermore, »32% of infants who survive GBS meningitis have neurodevelopmental impairment 18 months after illness, including 18% who have moderate-to-severe neurodevelopmental impairment (*6*). GBS is also a major cause of preterm delivery, stillbirths, and puerperal sepsis (*5**,**7*).

Screening pregnant women for GBS and offering intrapartum antimicrobial drug prophylaxis (IAP) to those who are found to be colonized, or have risk factors, has been widely implemented in many countries (*8*). However, the increased use of antimicrobial drugs has raised concerns regarding the emergence of resistance (*9*). Clindamycin and erythromycin resistance rates have increased greatly in the past 20 years (*10*) but might vary by geographic location (*10**,**11*). Knowledge of local antimicrobial drug resistance of GBS strains can contribute to optimal prophylactic and treatment strategies.

On the basis of the polysaccharide capsule, GBS strains are classified into 10 serotypes (*12*). A global review showed that serotype III was the most frequent isolate from infants who had invasive disease (*4*). Serotyping is of particular relevance to GBS vaccine development because most current candidates include serotype-specific polysaccharide–protein conjugate vaccines (*13*). An effective vaccine will need to prevent most infant disease, avoid the limitations of IAP, and cost-effective. Therefore, knowledge of prevalent serotypes will be relevant to country-specific decisions for vaccine implementation.

Evidence regarding the burden of invasive GBS disease in infants in China is limited. The recent systematic review found only 5 studies from China and estimated an incidence of 0.42 cases/1,000 live births for eastern Asia (*4*). This review was limited because it did not include publications in Mandarin Chinese and might not provide an accurate estimate of the burden of GBS disease in China. Therefore, we performed a systematic review and meta-analysis on the incidence, case-fatality rate (CFR), isolate antimicrobial resistance (AMR) patterns, and serotype and sequence type distributions for invasive GBS disease cases in infants <1–89 days of age in China.

## Methods

This systematic review was reported according to the Preferred Reporting Items for Systematic Reviews and Meta-Analyses guidelines (*14*). We focused on infants <1–89 days of age who had invasive GBS disease. We included studies that reported incidence and deaths associated with invasive disease, and antimicrobial drug resistance, serotypes, and multilocus sequence typing (MLST) of GBS isolates. Eligible studies were those published during January 1, 2000–March 16, 2018. The geographic scope of analysis was limited to China and included Taiwan, Hong Kong, and Macau.

### Definitions

Invasive GBS disease was defined as a positive GBS culture from any normally sterile site accompanied with signs of clinical disease. Early onset of GBS (EOGBS) was defined as isolation of GBS from infants <1–6 days after birth, and late onset of GBS (LOGBS) was defined as isolation of GBS from infants 7–89 days after birth. Incidence was defined as cases/1,000 live births (invasive GBS disease cases divided by live births at the respective hospital). CFR was defined as number of fatal GBS cases divided by total number of GBS cases. We categorized studies as prospective (data collected for the infant at admission and in hospital) and retrospective (data collected after the infant was discharged from a hospital).

In mainland China, hospitals were classified as primary, secondary, or tertiary institutions. A primary hospital is typically a township hospital that has <100 beds. These hospitals are tasked with providing preventive care, minimal healthcare, and rehabilitation services. Secondary hospitals tend to be affiliated with a medium-size city, county, or district and have >100 but <500 beds. These hospitals are responsible for providing comprehensive health services, as well as medical education and conducting research on a regional basis. Tertiary hospitals are comprehensive or general hospitals at the city, provincial, or national level that have >500 beds. These hospitals provide specialist health services, perform a larger role with regard to medical education and scientific research, and serve as medical hubs providing care to multiple regions.

### Search Strategy and Selection Criteria

We searched the PubMed/Medline, Embase, China National Knowledge Infrastructure, and Wanfang med online databases for literature published during January 1, 2000–March 16, 2018. We used the search terms “Streptococcus Group B” or “Group B streptococcal” OR “Streptococcus agalactiae” (medical subject headings) AND “infant,” “outcome,” “death,” “mortality,” “case AND fatality AND rate” for English databases. We used search terms “Group B streptococcal” OR “Streptococcus agalactiae” OR “GBS” AND “infant” OR “neonatal” in Chinese for Chinese databases. We limited searches to China, including Taiwan, Hong Kong, and Macau. An additional search for serotype data used the search terms “Streptococcus Group B serotype” or “Group B streptococcal serotype” OR “Streptococcus agalactiae serotype” (medical subject headings) and was performed with the same limits as listed above. We provide the full search strategy (Appendix Tables 1, 2).

We used snowball searches of article reference lists, including reviews, to identify additional studies. Two independent reviewers (Y.D. and Y.H.) critically appraised each paper and discussed discrepancies with a third coauthor (P.H.). We screened titles and abstracts according to specified inclusion and exclusion criteria, and then selected the full texts, followed by the details as described below.

### Inclusion and Exclusion Criteria

We included studies with original data on GBS invasive disease in infants <1–89 days of age, which had a population denominator (as the total number of live births at the respective hospital), CFR, serotype, or AMR. We provide full details of inclusion and exclusion criteria (Appendix Table 3).

### Data Abstraction and Quality Assessment

Isolates obtained from all normally sterile sites (blood, cerebrospinal fluid [CSF], lung aspirate, and joint specimens) were included for incidence estimates. For AMR, serotype, and MLST data, only isolates obtained from blood or CSF cultures were included. The quality of included studies was assessed in accordance with the Cochrane Handbook (*15*), including 9 items considered essential for good reporting of prevalence studies. Two independent reviewers (Y.D. and Y.H.) critically appraised each study. Disagreements were resolved by discussion with the third reviewer (P.H.).

### Statistical Analysis

We performed a meta-analysis by using Stata software version 14.0 (StataCorp, https://www.stata.com). We estimated overall incidence, EOGBS, LOGBS incidence, and CFR of GBS with random-effects meta-analyses by using the DerSimonian and Laird method. The Q test was performed to test heterogeneity between studies, and the *I^2^* was used to assess the degree of variation across studies. The level of heterogeneity was defined as low (*I^2^* = 25%), moderate (*I^2^* = 50%), and high (*I^2^* = 75%) (*15*). When heterogeneity was high, we also performed subgroup analysis based on study design (retrospective and prospective), isolate type (blood, CSF, and all sterile sites), and age of onset (EOGBS and LOGBS). Sensitivity analysis was conduct by excluding studies from Taiwan, Hong Kong, and Macau. As we anticipated, different infectious disease patterns, antimicrobial drug resistance, and healthcare systems in these regions might affect the estimates of GBS incidence and CFR. Potential publication bias was assessed by using a funnel plot and the Egger regression test. Descriptive analysis was performed to investigate the distribution of serotype and MLST typing. Antimicrobial drug resistance rates were reported by median with interquartile intervals.

## Results

### Literature Search and Study Selection

**Figure 1:**
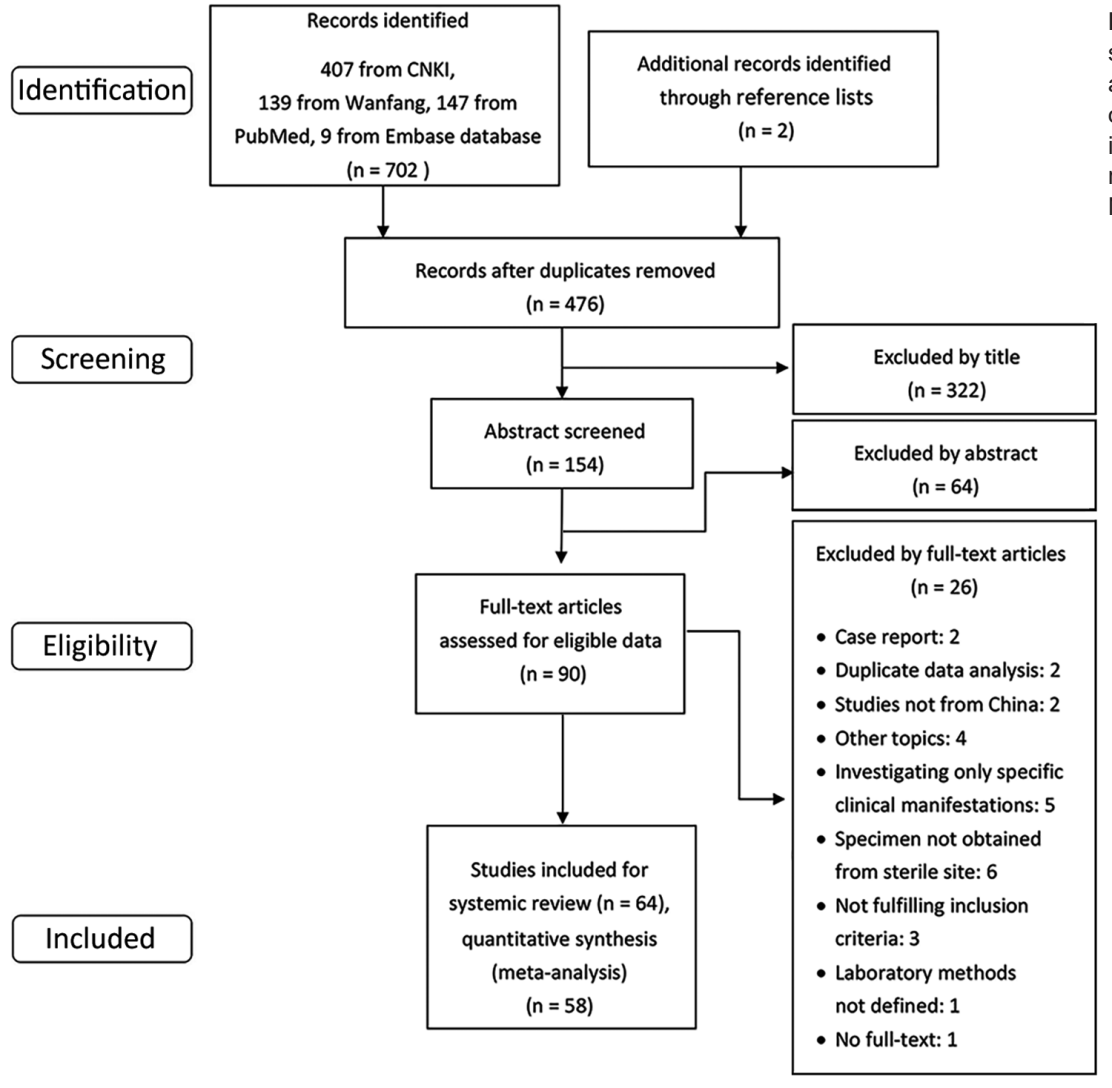
Process of study selection of systematic review and meta-analyses of incidence of group B *Streptococcus* disease in infants and antimicrobial resistance, China. CNKI, China National Knowledge Infrastructure.

We identified 704 published studies from database searches (407 from China National Knowledge Infrastructure, 139 from Wanfang, 147 from PubMed, and 9 from Embase). Two additional articles were identified from reference lists. A total of 64 articles met our inclusion criteria and search strategy (Figure 1). A total of 14 articles reported incidence, 56 articles reported CFR, 20 articles reported AMR, 4 articles reported serotype, and 2 articles reported MLST. We provide a full list of articles included (Appendix Table 4) and of articles excluded (Appendix Table 5). We provide the publication years of included studies (Appendix Figure 1).

### Study Characteristics

**Figure 2:**
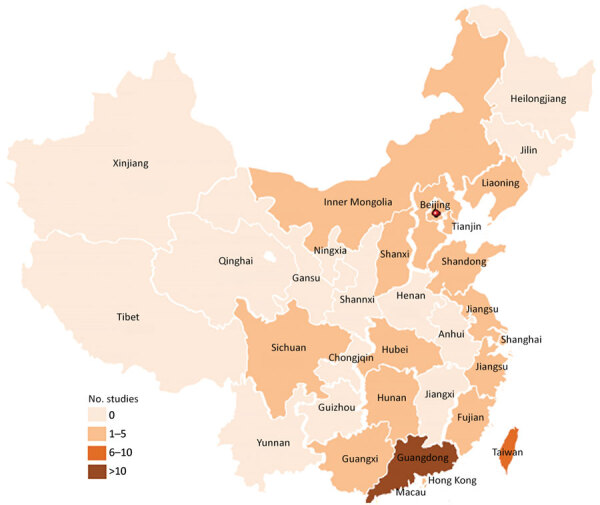
Distribution of study locations in systematic review and meta-analyses of incidence of invasive group B *Streptococcus* disease, by province, China.

Of the 64 studies included, 55 were from mainland China, 7 from Taiwan, 1 from Hong Kong, and 1 from Macau. On the basis of economic divisions, 92.2% (59/64) of studies were from eastern China, 2 each were from western and central China, and 1 was from northeastern China. Among the 55 articles from mainland China, 45 were from tertiary hospitals, 9 from secondary hospitals, and 1 from a primary hospital. The 7 articles from Taiwan and the 1 article from Hong Kong were all from teaching hospitals, and the 1 article from Macau was from a general hospital. We provide the distribution of studies of invasive GBS disease reported in China by province (Figure 2).

Among the 14 studies reporting incidence, 13 were from eastern China, and 1 from western China. Six (42.9%) of 14 papers reported use of IAP, all from eastern China; 3 (50%) of 6 IAPs were based on screening. Of the 56 studies that reported CFRs, 52 articles were from eastern China and 2 each were from central and western China. A total of 20 studies reported AMR, 19 papers from eastern China and 1 from northeastern China. Serotypes were available from 4 studies, all of them from eastern China. Only 2 articles included data on MLST. We provide characteristics of included studies and outcome types (Table 1). We also provide the risk for bias of the studies (Appendix Figure 2).

**Table 1:** Characteristics of included studies and outcome types for systematic review and meta-analyses of incidence of group B *Streptococcus* disease in infants, China*

Characteristic	Type and no. studies					
	Total, 64	Incidence, 14	CFR, 56	AMR, 20	Serotypes, 4	MLST, 2
China						
Eastern	59	13	52	19	4	2
Central	2	0	2	0	0	0
Western	2	1	2	0	0	0
Northeastern	1	0	0	1	0	0
Hospital type						
Mainland China						
Tertiary	45	6	39	18	4	2
Secondary	9	3	9	2	0	0
Primary	1	0	1	0	0	0
Nonmainland China						0
Teaching	8	4	7	0	0	0
General	1	1	0	0	0	0
Study design						
Prospective	4	3	3	0	1	1
Retrospective	60	11	53	20	3	1
Reporting period, days						
Full, 0–89	53	11	46	16	4	2
Full EOGBS <1–6	7	3	6	2	0	0
Full LOGBS 7–89	4	0	4	2	0	0
Specimen type						
Blood only	25	5	18	8	2	0
CSF only	6	0	6	3	0	0
Blood and CSF	23	6	22	9	2	2
All sterile sites	4	3	3	0	0	0
Blood and CSF plus sputum or gastric fluid	6	0	7	0	0	0
IAP						
Any	10	6	9	3	1	1
None	4	0	3	2	0	0
Unknown	50	8	44	15	3	1

*AMR, antimicrobial resistance; CFR, case-fatality rate; CSF, cerebrospinal fluid; EOGBS, early onset group B *Streptococcus*; IAP, intrapartum antimicrobial drug prophylaxis; MLST, multilocus sequence typing; LOGBS, late onset group B *Streptococcus*.

### Incidence of Invasive GBS Disease

**Figure 3:**
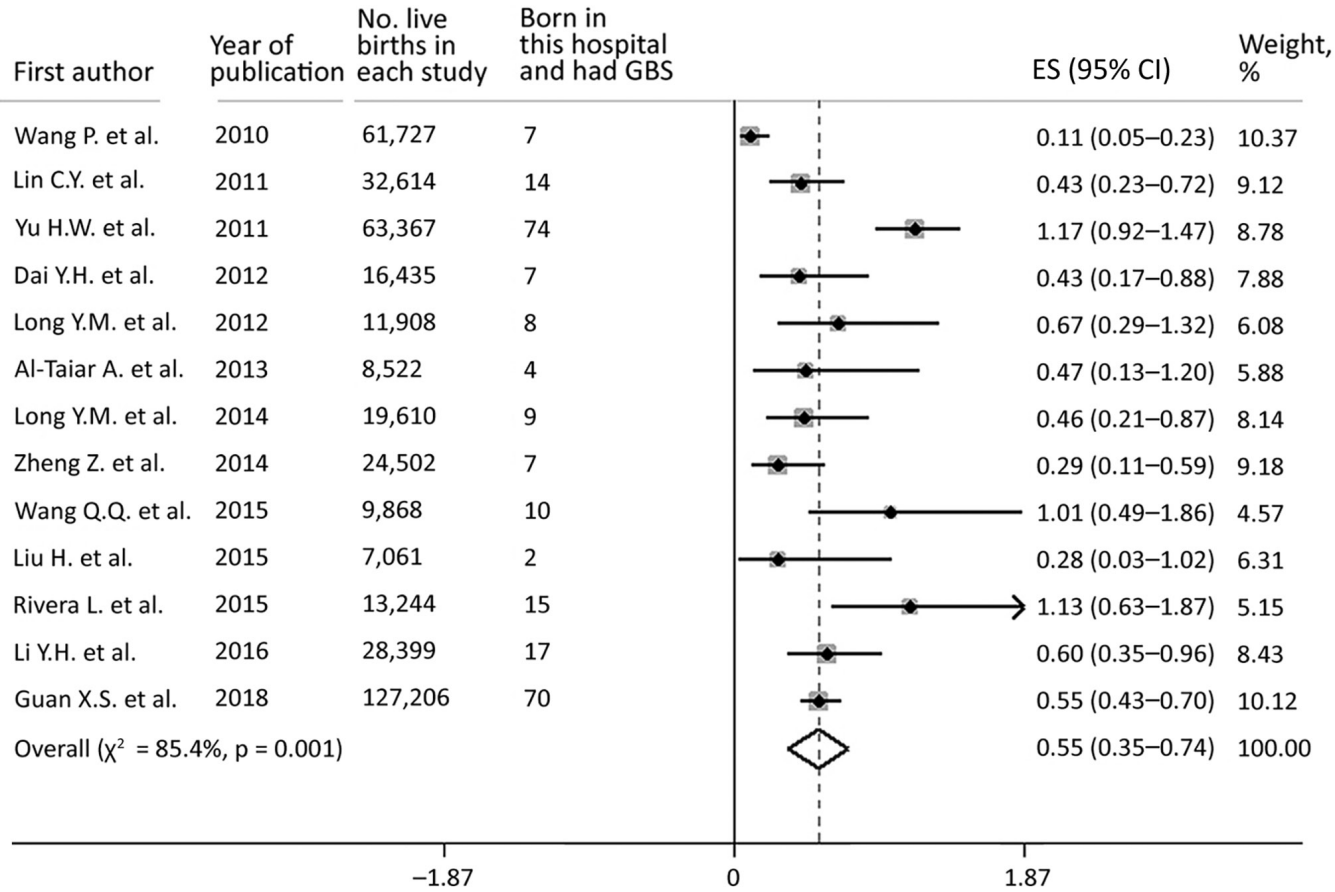
Overall incidence risk per 1,000 live births of invasive GBS disease in 13 infants <1–89 days of age, China. Vertical dashed line indicates a visual assessment of heterogeneity of the studies. If the vertical line can be drawn, forest plots indicate that all studies are similar enough to be included for meta-analysis. Error bars indicate 95% CIs. Reference details are provided in the Appendix. ES, effect size; GBS, group B *Streptococcus* disease.

Of the 14 relevant studies, 13 reported raw data on live births, which enabled a meta-analysis to be performed. Of 424,463 live births, 244 infants had invasive GBS disease at the age of 0–89 days; the pooled estimated incidence was 0.55 cases/1,000 live births (95% CI 0.35–0.74 case/1,000 live births). Significant heterogeneity was observed (p = 0.0001, *I^2^* = 85.4%) (Figure 3). Subgroup analyses were conducted to assess heterogeneity by study design, isolate site, and age of onset. Among the 13 studies reporting raw data on live births, 11 studies distinguished early-onset and late-onset cases (n = 3 studies) born in a hospital. There were 133 cases of EOGBS for 352,574 live births, an incidence of 0.38 cases/1,000 live births (95% CI 0.25–0.51 cases/1,000 live births), and 33 cases of LOGBS for 168,849 live births, an incidence of 0.18 cases/1,000 live births (95% CI 0.11–0.25 cases/1,000 live births). We provide results of meta-analysis for LOGBS incidence (Appendix Figure 3), for EOGBA incidence (Appendix Figure 4), and for subgroup analyses (Appendix Table 6).

Sensitivity analysis was conducted to confirm the stability and liability of the meta-analysis by excluding data for Taiwan, Hong Kong, and Macau. This exclusion resulted in a pooled incidence of invasive GBS disease of 0.44 cases/1,000 live births (95% CI 0.25–0.63 cases/1,000 live births) for mainland China (Appendix Figure 5). According to the funnel plot and p value of the Eggers regression test (p = 0.069 [>0.05]), there was no visually apparent publication bias of included studies (Appendix Figure 6).

### CFRs for GBS Invasive Disease

A total of 56 papers reported CFR data for infants <1–89 days of age. Of 1,439 infants with GBS invasive disease, 106 died. The overall pooled estimated CFR rate was 5.0% (95% CI 3.0%–6.0%). The EOGBS CFR was 6.0% (4.0%–8.0%), and LOGBS CFR was 4.0% (1.0%–6.0%). We provide results of meta-analysis for overall, EOGBS, and LOGBS CFRs (Appendix Figures 7, 8, and 9, respectively). Sensitivity analysis was conducted to confirm the stability and liability of the meta-analysis by including only studies from mainland China. The pooled estimated CFR was 4.0% (95% CI 2%.0%–6.0%) when data for only mainland China were included (Appendix Figure 5).

### Antimicrobial Resistance

A total of 20 articles reported antimicrobial resistance for 598 GBS isolates. The highest prevalence of resistance was reported for tetracycline (median 98.0%, interquartile range [IQR] 80.0%–100%), followed by clindamycin (73.3%m IQR 62.6%–78.7%), erythromycin (64.4%, IQR 56.6%–75%), and ciprofloxacin (25.0%, IQR 9.1%–35.2%). There was no reported resistance to penicillin, ampicillin, vancomycin, or linezolid. For ceftriaxone, the median prevalence of resistance was 0% (IQR 0%–60.0%), although 1 study reported 100% prevalence of resistance (1/1 isolates), and 1 study reported 80% resistance (12/15 isolates) (Table 2).

**Table 2:** Proportion of isolates demonstrating antimicrobial resistance in systematic review and meta-analyses of incidence of group B *Streptococcus* disease in infants, China*

Reference	Publication year	No. isolates	PEN	AMP	CFZ	CAX	VAN	LZD	CHL	ERY	TET	CIP	MXF	LVX	NIT	TGC
Zeng et al.	2013	11	0	0	NT	NT	0	0	NT	NT	100.0	9.1	9.1	9.1	0	0
Luo et al.	2013	15	0	0	NT	0	0	NT	NT	86.7	NT	NT	NT	0	NT	NT
Zheng et al.	2014	12	0	0	NT	NT	0	NT	NT	16.7	66.7	NT	NT	NT	NT	NT
Chen et al.	2014	16	0	0	NT	0	0	NT	NT	62.5	NT	25.0	NT	18.8	NT	NT
Zhu et al.	2014	13	0	10.0	0	100.0	38.5	0	100.0	100.0	NT	33.3	NT	8.3	0	NT
Fan et al.	2014	42	0	0	NT	NT	0	0	NT	69.1	73.8	NT	NT	38.1	NT	0
Wang et al.	2015	15	0	20.0	40.0	80.0	0	0	86.7	100.0	NT	26.7	NT	20.0	0	NT
Zhang et al.	2015	6	0	0	83.3	NT	0	0	NT	NT	NT	NT	NT	NT	NT	NT
Lei et al.	2015	20	NT	0	NT	NT	0	0	25.0	75.0	NT	80.0	NT	70.0	0	NT
Zhang et al.	2015	45	0	2.2	NT	NT	0	0	NT	42.2	93.3	0	0	0	NT	0
Cai et al.	2016	15	0	0	NT	NT	0	0	NT	46.7	100.0	NT	6.7	6.7	13.3	0
Zhao	2016	28	0	0	NT	0	0	3.6	NT	67.9	NT	NT	NT	42.9	NT	NT
Huang et al.	2016	49	NT	NT	NT	NT	NT	NT	NT	63.3	98.0	11.9	12.2	7.7	NT	NT
Liu et al.	2017	15	0	NT	NT	NT	0	NT	NT	NT	NT	NT	NT	NT	NT	NT
Zhang et al.	2017	55	0	0	NT	NT	0	0	NT	56.6	98.1	1.9	NT	NT	NT	NT
Zhang et al.	2017	15	6.7	0	NT	NT	0	0	NT	NT	80.0	73.3	73.3	60.0	0	0
Tan et al.	2017	20	0	0	NT	NT	0	0	NT	NT	100.0	16.7	NT	16.7	NT	0
Zhou et al.	2017	84	4.8	2.4	2.4	0	0	0	4.8	72.6	100.0	35.2	NT	36.9	0	NT
Zhao	2017	45	0	NT	NT	0	0	2.2	NT	64.4	NT	NT	NT	42.2	NT	NT
Guan et al.	2018	68	0	NT	NT	0	0	0	NT	57.4	95.6	NT	NT	5.9	NT	NT
Median	NA	NA	0	0	21.2	0	0	0	55.8	64.4	98.0	25.0	9.1	17.7	0	0
IQI 25%	NA	NA	0	0	0.6	0	0	0	9.8	56.6	80.0	9.1	3.3	6.9	0	0
IQI 75%	NA	NA	0	1.7	72.5	60.0	0	0	96.7	75.0	100	35.2	42.8	41.2	0	0

*Values are percentages. Reference details are provided in the Appendix (https://wwwnc.cdc.gov/EID/26/11/18-1414-App1.pdf). Green indicates a rate of AMR <25%; yellow 25%–50%; red >50%; 25%, and 75% refers to AMR interquartile interval of 25% and 75%. Amp, ampicillin; CFZ, cefazolin; CAX, ceftriaxone, CHL, chloramphenicol; CIP, ciprofloxacin; ERY, erythromycin; IQI, interquartile interval; LZD, linezolid; MXF, moxifloxacin; NA, not applicable; NIT, nitrofurantoin; NT, not tested; PEN, penicillin: TET, tetracycline; TGC, tigecycline; VAN, vancomycin.

### Serotype Distribution

Four studies included data on serotypes for 175 invasive GBS cases. All of these studies were from eastern China. Four serotypes (Ia, Ib, III, and V) accounted for 97% of invasive isolates. Serotype III was the most common (65%, 114/175), followed by Ib (16%, 27/175), Ia (10%, 18/175), and V (6%, 11/175). Two articles distinguished EO and LOGBS serotypes; there were 24 EOGBS isolates and 52 LOGBS isolates. Serotype III predominated in both EO (15/24, 63%) and LOGBS (40/52, 77%) (Appendix Figure 10).

### MLST

Only 2 studies reported MLST. Of 76 isolates 15 sequence types (STs) were reported. A total of 89% (68/76) of strains belonged to 6 STs (ST17, ST12, ST23, ST1, ST19, and ST650). More than half (58%, 44/74) of the samples were ST17, followed by ST12 (9%, 7/76) and ST23 (7%, 5/76); ST1, ST19, and ST650 each accounted for 5% (4/76).

### Relationship between Serotype and MLST

Only 2/76 papers included data on serotype and MLST. A total of 80% (44/55) of serotype III strains were shown to be ST17, and 54% (7/13) of serotype Ib strains were ST12 (Appendix Table 7).

## Discussion

The annual number of births in China ranged from 15.7 million to 17.8 million between 2001 and 2016 (*16*). Thus, with an estimated pooled incidence of 0.55 cases/1,000 live births (95% CI 0.35–0.74 cases/1,000 live births), there is a substantial burden of invasive GBS disease for infants in China. This incidence is also higher than that for all infants in the recent global review (0.49 cases/1,000 live births, 95% CI 0.43–0.56 cases/1,000 live births) and higher than that previously defined for eastern Asia (0.42 cases/1,000 live births) (*4*). Unlike most industrialized countries, there are no national guidelines for GBS screening and prevention in China, although in 43% of studies from China, IAP was mentioned. However, there are no data on the extent to which IAP is currently used in China. Previous studies suggest that the low incidence of GBS infection for infants in Asia might be related to a lower rate of GBS colonization in pregnant women (*17*). A review of colonization identified 30 studies from China, which included 44,716 women and showed an overall colonization rate of 11.3%. However, several studies from China reported much higher rates of GBS colonization (31%–36%) (*18*,*19*), suggesting substantial variability.

The CFR in our study (5.0%, 95% CI, 3.0%–6.0%) was lower than that estimated from the global review (8.4%, 95% CI 6.6%–10.2%) (*4*). Most of our data were for level-3 teaching hospitals in which use of antimicrobial drugs and standard of medical care might be higher, which might explain a lower mortality rate. We do not have information on birthweight and gestational age of infants with GBS disease with which we can compare with other settings; the CFR for preterm infants is known to be much higher (*1*).

The prevalence of resistance to clindamycin and erythromycin appear to be high in China. A study in Canada showed the prevalence of resistance to clindamycin was 4.5% and to erythromycin was 8% (*9*). In England and Wales, erythromycin resistance in isolates causing disease in infants was 15% for EO disease and 13% for LO disease (*20*). In South Korea, the prevalence of resistance to erythromycin was 42.9%–51.8% and for clindamycin was 55.4% (*11*,*21*), suggesting that the prevalence might be much higher in Asia. This finding is consistent with systematic review (*22*) of GBS isolates causing colonization that reported a pooled prevalence of resistance of 25% for erythromycin and 27% for clindamycin, and notably higher prevalences in Asia (46% for erythromycin and 47% for clindamycin). A study of colonization of pregnant women in China also reported that most isolates were resistant to tetracycline (76.9%), erythromycin (72.1%) and clindamycin (66.4%) (*23*). Macrolide resistance in streptococci is caused mainly by a macrolide–specific efflux mechanism encoded by the *mef* A gene and ribosomal modification by a methylase associated with *erm* (erythromycin ribosome methylase) genes (*24*,*25*). Erythromycin resistance was associated mainly with *ermB* and *mef (A/E)* genes in China (*26*,*27*). The *erm(B)* and *erm(TR/A)* genes were the main macrolide-resistant genes in Spain and Canada (*9*,*25*), and *erm B* and *lnuB* genes were prevalent in South Korea (*28*).

Resistance to erythromycin and clindamycin presents a challenge for treatment and prophylaxis strategies because these antimicrobial drugs are often used for patients in China who are allergic to penicillin. However, GBS isolates were susceptible to penicillin, ampicillin, vancomycin, and linezolid, consistent with other reports (*9**,**21**,**22*). The apparent resistance to ceftriaxone is unusual and, as noted, the sample size for these 2 studies was small. Furthermore, because no details were provided on the methods used for testing the isolates, it is essential that this reported resistance is verified.

The serotype and MLST distribution of invasive GBS disease isolates in China is consistent with the global review (*4*); serotype III and ST17 are the most prevalent types (*21*,*29*). Therefore, our data suggest that a conjugate vaccine incorporating 5 serotypes (III, Ia, Ib, II, and V) could cover 97% of invasive GBS disease in infants <3 months of age in China.

Currently, there is limited evidence on the burden of GBS disease for infants in China. Our comprehensive review is a major addition to the literature because it includes a systematic review of studies in the Chinese language, as well as data on incidence, antimicrobial drug susceptibility, and MLST types.

There are several potential limitations to this study. First, major heterogeneity among studies was observed. Although potential sources of heterogeneity were explored by subgroup analyses, none of them sufficiently explain the heterogeneity. Sensitivity analysis suggests that the pooled estimated incidence and CFR was changed when Taiwan, Hong Kong, and Macau were excluded. This finding is plausible and might reflect the differences in healthcare systems compared with those of mainland China. Second, we did not search for unpublished studies, which could result in publication bias. Third, we were not able to assess the time of sample collection or the methods of collection, culture, and antimicrobial drug sensitivity testing. Fourth, there were limited data available on serotypes and MLST types; thus, meta-analysis was not possible. Fifth, for CFRs, we were only able to include patients who died in a hospital; thus, the true CFR might be higher.

The estimated burden of infant GBS disease in China is substantial, suggesting that implementation of additional prevention efforts could be beneficial. Interventions to be considered could include a coordinated national strategy for maternal GBS screening with administration of intrapartum antimicrobial drug prophylaxis, and, when available, maternal vaccination with an effective GBS vaccine. Further research to clarify the noted heterogeneity in infant GBS disease in China, as well as research to assess the acceptability, logistics, and cost-effectiveness of maternal GBS vaccination could help guide these eff­orts.

Appendix Additional information on systematic review and meta-analyses of incidence for group B *Streptococcus* disease in infants and antimicrobial resistance, China.
