# Supplementary material for: Systematic Review and Meta-Analyses of Incidence for Group B Streptococcus Disease in Infants and Antimicrobial Resistance, China
Source: Emerg Infect Dis. 2020 Nov;26(11):2651–9. doi: 10.3201/eid2611.181414 (PMC7588546; doi:10.3201/eid2611.181414)
Supplement: Appendix — Additional information on systematic review and meta-analyses of incidence for group B Streptococcus disease in infants and antimicrobial resistance, China. [file 18-1414-Techapp-s1.pdf]

Article DOI: https://doi.org/10.3201/eid2611.181414

Systematic Review and Meta-Analyses of Incidence for Group B *Streptococcus* Disease in Infants and Antimicrobial Resistance, China

Appendix

Appendix Table 1. Search terms (for English papers) and search period (January 1, 2000–March 16, 2018) for PubMed/ Medline or Embase (search date: March 17, 2018)*

Search term
Infant
Outcome
Death
Mortality
Case AND Fatality AND rate
Death [MeSH terms]
Mortality [MeSH terms]
Case fatality rate [MeSH terms]
AND
Streptococcal
Streptococcus
Streptococci AND (Group AND B) or agalactiae
Streptococcus agalactiae [MeSH terms]
AND
Streptococcus serotype
Streptococcal serotype
Streptococcus agalactiae serotype [MeSH terms]

*MeSH, medical subject headings

Appendix Table 2. Search terms (for Chinese papers) and search period (January 1, 2000–March 16, 2018) for China National Knowledge Infrastructure or Wanfang med online databases (search date: March 18, 2018)

Search term
族 (Group B Streptococcal)
无乳 (*Streptococcus agalactiae*)
AND
新生儿 (Neonatal)
(Infant)
AND
血清型 (Serotype)

Appendix Table 3. Inclusion and exclusion criteria*

Characteristic	Inclusion criteria	Exclusion criteria
Population	Invasive GBS disease in infants <1–89 days of age at onset of infection	Studies containing only information on high-risk groups
Laboratory	GBS confirmed by blood, CSF, or other sterile site culture	NA
Search	No language restrictions	Foreign language papers for which it was not possible to obtain English or Chinese translations
Study	Study reporting more recent data from country or hospital	Case report, case series, reviews, conference papers; studies from the same country or hospital reporting repeated years or data.

*CSF, cerebrospinal fluid; GBS, group B *Streptococcus*; NA, not applicable.

Appendix Table 4. Characteristics of included studies for infant invasive group B *Streptococcus* (GBS) disease in children*

Reference	Region of China	Year of publication	Year of data collection	Incidence	CFR	AMR	Serotype	MLST	IAP	Reporting period, y	Study design
Chang CJ et al. (*1*)	Taiwan	2003	1986.1–2001.12	N	Y	N	N	N	U	<1–89	R
Chung MY et al. (*2*)	Taiwan	2004	1996.1.1–2002.12.31	Y	Y	N	N	N	U	<1–89	R
Jiang JH et al. (*3*)	Taiwan	2004	1992.1–2001.12	N	Y	N	N	N	N	<1–89	R
Wu JH et al. (*4*)	Taiwan	2009	2001.1–2006.12	N	Y	N	N	N	U	<1–89	P
Wang P et al. (*5*)	Beijing	2010	2005–2009	Y	Y	N	N	N	U	<1–6	R
Liu ZW et al. (6)	Shang Hai	2011	1999.1–2008.12	N	Y	N	N	N	U	<1–89	R
Lin CY et al. (7)	Taiwan	2011	2001.1–2008.11	Y	N	N	N	N	Y	<1–6	R
Yu HW et al. (*8*)	Taiwan	2011	2002.1–2005.6	Y	Y	N	N	N	Y	<1–89	R
Wu MF (*9*)	Guang Dong	2012	2008.1–2012.1	N	Y	N	N	N	U	<1–89	R
Dai YH et al. (*10*)	Guang Dong	2012	2008.6–2011.4	Y	Y	N	N	N	U	<1–89	R
Long YM et al. (*11*)	Guang Dong	2012	2009.7–2011.6	Y	Y	N	N	N	U	<1–89	R
Zeng SJ et al. (*12*)	Guang Dong	2013	2012.1–2012.12	N	Y	Y	N	N	U	<1–89	R
Luo J et al. (*13*)	Guang Dong	2013	2007.1–2011.12	N	Y	Y	N	N	U	7–89	R
Chen L et al. (*14*)	Guang Dong	2013	2010–2012	N	Y	N	N	N	U	<1–89	R
Al-Taiar A et al. (*15*)	Macau	2013	2006.1.1–2009.12.31	Y	N	N	N	N	U	<1–89	P
Wu YY (*16*)	Guang Dong	2014	2010–2013	N	Y	N	N	N	U	<1–89	R
Fan WH et al. (*17*)	Beijing	2014	2011.1–2013.9	N	N	Y	N	N	U	<1–89	R
Zheng Z et al. (*18*)	Fujian	2014	2011.10–2013.4	Y	Y	Y	N	N	Y	<1–6	R
Chen Y et al. (*19*)	Guang Dong	2014	2011.1–2013.10	N	Y	Y	N	N	U	<1–6	R
Wei CP et al. (*20*)	Shan Dong	2014	2012–2014	N	Y	N	N	N	U	<1–89	R
Huang HJ et al. (*21*)	Guang Dong	2014	2011.1–2012.12	N	Y	N	N	N	U	<1–89	R
Long YM et al. (*22*)	Guang Dong	2014	2011.1–2013.12	Y	Y	N	N	N	U	<1–89	R

Reference	Region of China	Year of publication	Year of data collection	Incidence	CFR	AMR	Serotype	MLST	IAP	Reporting period, y	Study design
Zhu ML et al. (23)	Zhe Jiang	2014	2005.1–2013.5	N	Y	Y	N	N	Y	<1–89	R
Liu X et al. (*24*)	Jiang Su	2015	2013.3–2015.3	N	Y	N	N	N	U	<1–89	R
Zhang S et al. (*25*)	Guang Dong	2015	2013.1–2014.3	N	Y	Y	N	N	U	7–89	R
Zeng SJ et al. (*26*)	Guang Dong	2015	2012–2014	N	N	N	Y	N	U	<1–89	R
Li K et al. (*27*)	Guang Dong	2015	2011.3–2014.2	N	Y	N	N	N	U	<1–89	R
Wang QQ et al. (*28*)	Zhe Jiang	2015	2010.4–2014.4	Y	Y	Y	N	N	Y	<1–89	R
Wang YC et al. (*29*)	Jiang Su	2015	2013.1–2013.12	N	Y	N	N	N	Y	<1–89	R
Luo MJ et al. (*30*)	Guang Dong	2015	2010–2012	N	Y	N	N	N	U	<1–6	R
Zhao N et al. (*31*)	Guang Dong	2015	2011.11–2014.4	N	Y	N	N	N	U	<1–89	R
Lei MF et al. (*32*)	Tianjin	2015	2006.12.-2014.09	N	Y	Y	N	N	U	<1–89	R
Liu H et al. (*33*)	Guang Dong, Hunan	2015	2013.09–2014.09	Y	Y	N	Y	Y	Y	<1–89	P
Rivera L et al. (*34*)	Hong Kong	2015	U	Y	Y	N	N	N	Y	<1–89	P
Zhang JS et al. (*35*)	Guang Dong	2015	2010–2014	N	Y	Y	N	N	U	<1–89	R
Liu ZY et al. (*36*)	Fu jian	2016	2011.3–2014.10	N	Y	N	N	N	U	<1–89	R
Zhang XH et al. (*37*)	Shan Xi (Tai Yuan)	2016	2013.1–2015.11	N	Y	N	N	N	U	<1–89	R
Li L et al. (*38*)	Guang Dong	2016	2008.1–2014.8	N	Y	N	N	N	U	<1–89	R
Li YH et al. (*39*)	Nei Menggu	2016	2013.6–2016.6	Y	Y	N	N	N	U	<1–89	R
Yang HH et al. (*40*)	Shang Hai	2016	2012.1–2015.5	N	Y	N	N	N	N	<1–89	R
Shen YH et al. (*41*)	Beijing	2016	2008.1–2014.1	N	Y	N	N	N	U	<1–89	R
Cai YF et al. (*42*)	Guang Dong	2016	2011.1–2014.10	N	Y	Y	N	N	U	<1–89	R
Lai JD et al. (*43*)	Fu Jian	2016	2010.1–2015.2	N	Y	N	N	N	U	<1–6	R
Zhao L (*44*)	Jiang Su	2016	2014.4–2016.4	N	Y	Y	N	N	U	<1–89	R
Ju HQ et al. (*45*)	Shang Hai	2016	2010.3–2015.2	N	Y	N	N	N	U	<1–89	R
Huang LF et al. (*46*)	Guang Dong	2016	2010.11–2014.2	N	N	Y	N	N	U	<1–89	R

Reference	Region of China	Year of publication	Year of data collection	Incidence	CFR	AMR	Serotype	MLST	IAP	Reporting period, y	Study design
Yue D (*47*)	Hu Bei	2017	2014.1–2016.1	N	Y	N	N	N	U	<1–89	R
Qiao LY et al. (*48*)	Shan Dong	2017	2012.1–2016.1	N	Y	N	N	N	U	7–89	R
Guan XS et al. (*49*)	Guang Dong	2017	2012.1–2015.12	N	Y	N	N	N	U	<1–89	R
Liu WW et al. (*50*)	Guang Dong	2017	2012.1–2015.12	N	Y	Y	N	N	U	<1–89	R
Lv CH (*51*)	Shan Dong	2017	2014.1–2015.12	N	Y	N	N	N	U	<1–89	R
Zhou YZ et al. (*52*)	Zhe Jiang	2017	2008.2–2016.11	N	N	Y	Y	N	U	<1–89	R
Zhang JS et al. (*53*)	Guang Dong	2017	2010.1.1–2015.21.31	N	Y	Y	N	N	U	<1–89	R
Zhang N et al. (*54*)	Shan Dong	2017	2013.1–2016.5	N	Y	Y	N	N	N	<1–89	R
Wang YJ et al. (*55*)	Guang Dong	2017	2011.4–2015.4	N	Y	N	N	N	U	7–89	R
Shenzhen GBS study group (*56*)	Guang Dong	2017	2010.1–2016.6	N	Y	N	N	N	Y	<1–89	R
Zhang S et al. (*57*)	Beijing	2017	2010–2014	N	Y	N	N	N	U	<1–89	R
Tan KH et al. (*58*)	Guang Dong	2017	2012.3–2016.3	N	N	Y	N	N	N	<1–89	R
Zhao TL (*59*)	Liaoning	2017	2015.1–2016.2	N	N	Y	N	N	U	<1–89	R
Ma HL et al. (*60*)	Si Chuan	2017	2014.1–2016.2	N	Y	N	N	N	U	<1–6	R
Huang W et al. (*61*)	Gong Dong, Guang Xi	2017	2013.1–2015.2	N	Y	N	N	N	U	<1–89	R
Chen IL et al. (*62*)	Taiwan	2017	2008.1–2013.12	N	Y	N	N	N	U	<1–89	R
Chen HY et al. (*63*)	Zhe Jiang	2018	2014.6.1–2017.6.31	N	Y	N	N	N	Y	<1–89	R
Guan XS et al. (*64*)	Guang Dong	2018	2011.1–2014.12	Y	Y	Y	Y	Y	U	<1–89	R

*AMR, antimicrobial drug resistance; CFR, case-fatality rate; GBS, group B *Streptococcus*; IAP, intrapartum antimicrobial drug prophylaxis; MLST, multilocus sequence typing; N, no; P, prospective study; R, retrospective study; U, unknown (information not available); Y, yes.

Appendix Table 5. Studies with reasons for exclusions

Reference	Year of publication	Year of data collection	Reasons for exclusion
Resiner DP et al. (*65*)	2000	1994.2–1997.1	Studies not from China
Chang C et al. (*66*)	2000	1984–1997	Investigating only specific clinical manifestations
Zhong Y et al. (*67*)	2002	1998.11–1999.7	Not fulfilling inclusion criteria
Liao CH et al. (*68*)	2002	1980.1–2000.3	No full text
Tiskumara R et al. (*69*)	2009	2005.1.1–2005.12.31	Studies not from China
Lin MC et al. (*70*)	2012	1984–2008	Investigating only specific clinical manifestations
Ye F et al. (*71*)	2013	2009–2011	Other topics
Zhang J et al. (*72*)	2013	2010.1–2011.1	Case report
Lin Z et al. (*73*)	2013	2009.1–2013.5	Investigating only specific clinical manifestations
Tan JF et al. (*74*)	2014	2011.8–2012.8	Other topics
Chu SM et al. (*75*)	2014	20014.1–2011.12	Other topics
Zhang J et al. (*76*)	2015	2009.1–2012.12	Duplicate data analysis
Li L et al. (*77*)	2015	2008.1–2014.8	Not fulfilling inclusion criteria
Mu L et al. (*78*)	2015	2011.7.2014.7	Specimen not obtained from sterile site
Zhong H et al. (*79*)	2015	2011–2014	Specimen not obtained from sterile site
Zhong H et al. (*80*)	2015	2011.1–2014.5	Duplicate data analysis
Wang P et al. (*81*)	2015	2008–2013	Not defined laboratory methods
Li L et al. (*82*)	2016	2008.1–2014.8	Not fulfilling inclusion criteria
Wang Y et al. (*83*)	2016	2013.9–2015.9	Specimen not obtained from sterile site
Geng H et al. (*84*)	2016	2010–2015	Other topics
Huang J et al. (*85*)	2016	2011.11–2015.9	Specimen not obtained from sterile site
Hua CZ et al. (*86*)	2016	2011.1–2015.12	Investigating only specific clinical manifestations
Ding Y et al. (*87*)	2017	2008–2015	Case report
Wang Y et al. (*88*)	2017	2015.10–2016.12	Specimen not obtained from sterile site
Jing L et al. (*89*)	2017	2009.1–2015.2	Specimen not obtained from sterile site
Wu IH et al. (*90*)	2017	2006.1–2013.12	Investigating only specific clinical manifestations

Appendix Table 6. Results of subgroup analysis of total incidence of GBS invasive disease*

			Heterogeneity test	
Subgroup	No. studies	Incidence (95% CI)	I2, %	Q test p value
Study design				
Retrospective	10	0.54 (0.32–0.75)	88.20	0.001
Prospective	3	0.60 (0.12–1.08)	56.80	0.10
Isolate type				
Blood	5	0.37 (0.14–0.60)	69.70	0.01
All sterile sites	1	1.17 (0.89–1.44)		
Blood plus CSF	7	0.52 (0.35–0.69)	46.00	0.09
Age of onset, y				
EOGBS	11	0.38 (0.25–0.51)	65.40	0.001
LOGBS	3	0.18 (0.11–0.25)	0.0	0.45

*CSF, cerebrospinal fluid; EOGBS; early-onset group B *Streptococcus*; LOGBS, late-onset group B *Streptococcus.*

Appendix Table 7. Relationship between group B *Streptococcus* serotypes and MLST results*

Author	No samples	Serotype	ST17	ST12	ST23	ST10	ST1	New 17-like
Liu H et al.	2	III	1	0	0	0	0	1
	3	Ib	0	2	0	1	0	0
	2	Ia	0	0	2	0	0	0
	1	V	0	0	0	0	1	0
Guan XS et al.	53	III	43	0	0	0	0	0
	10	Ib	0	5	1	1	0	0
	2	Ia	0		2	0	0	0
	3	V	0	0	0	0	3	0

*MLST, multilocus sequence typing; ST, sequence type.

References

1. Chang CJ, Chang WN, Huang LT, Huang SC, Chang YC, Hung PL, et al. Neonatal bacterial meningitis in southern Taiwan. Pediatr Neurol. 2003;29:288–94. PubMed https://doi.org/10.1016/S0887-8994(03)00273-X

2. Chung MY, Ko DJ, Chen CC, Huang CB, Chung CH, Chen FS, et al. Neonatal group B streptococcal infection: a 7-year experience. Chang Gung Med J. 2004;27:501–8. PubMed

3. Jiang JH, Chiu NC, Huang FY, Kao HA, Hsu CH, Hung HY, et al. Neonatal sepsis in the neonatal intensive care unit: characteristics of early versus late onset. J Microbiol Immunol Infect. 2004;37:301–6. PubMed

4. Wu JH, Chen CY, Tsao PN, Hsieh WS, Chou HC. Neonatal sepsis: a 6-year analysis in a neonatal care unit in Taiwan. Pediatr Neonatol. 2009;50:88–95. PubMed https://doi.org/10.1016/S1875-9572(09)60042-5

5. Wang P, Ma J, Wang Y, Wen C, Li H, Zhuang T, et al. Perinatal clinical features of early-onset neonatal septicemia caused by group B streptococcus. Clin J Neonatol. 2010;25:219–22.

6. Liu Z, Tang Z, Ding Y, Huang X. Study of early-onset and late-onset neonatal sepsis. J Clin Pediatr. 2011;29:446–9.

7. Lin CY, Hsu CH, Huang FY, Chang JH, Hung HY, Kao HA, et al. The changing face of early-onset neonatal sepsis after the implementation of a maternal group B *Streptococcus* screening and intrapartum prophylaxis policy: a study in one medical center. Pediatr Neonatol. 2011;52:78–84. PubMed https://doi.org/10.1016/j.pedneo.2011.02.001

8. Yu HW, Lin HC, Yang PH, Hsu CH, Hsieh WS, Tsao LY, et al. Group B streptococcal infection in Taiwan: maternal colonization and neonatal infection. Pediatr Neonatol. 2011;52:190–5. PubMed https://doi.org/10.1016/j.pedneo.2011.05.008

9. Wu M. Clinical analysis of 20 cases of neonatal group B streptococcal septicaemia. Contemp Med. 2012;18:70–1.

10. Dai Y, Zeng L, Gao P. Clinical analysis of 14 cases of neonatal group B streptococcal septicaemia. Clin J Neonatol. 2012;27:44–6.

11. Long Y, Zhang Z, Chen X. Clinical analysis of 13 cases of neonatal group B streptococcal septicaemia. China Health Care and Nutrition. 2012;9.

12. Zeng S, Qiu H. Clinical analysis of 11 cases of neonatal *Streptococcus agalactiae* sepsis. Zhongguo Fuyou Baojian. 2012;28:3290–1.

13. Luo J, Ma L, Xu F, Lu G, Feng Z. Clinical characteristics and prognosis of late-onset group B streptococcal sepsis in NICU. J Clin Pediatr. 2013;3:805–8.

14. Chen L, Wu B, Cheng H, Tang Y, Wu J, Yan X, et al. Group B Streptococcal septicemia combined with purulent meningitis: clinical analysis of five cases. Chin Gen Pract. 2013;16:2750–2.

15. Al-Taiar A, Hammoud MS, Cuiqing L, Lee JK, Lui KM, Nakwan N, et al. Neonatal infections in China, Malaysia, Hong Kong and Thailand. Arch Dis Child Fetal Neonatal Ed. 2013;98:F249–55. PubMed https://doi.org/10.1136/archdischild-2012-301767

16. Wu Y. Clinical analysis of 88 cases of neonatal group B hemolytic streptococcal infection. J Frontier Med. 2014;17:280–1.

17. Fan W, Zhao M, Liu J. Antimicrobial resistance in 42 cases of neonate septicemia caused by *Streptococcus agalactiae* infection. Int J Lab Med. 2014;35:2309–10.

18. Zheng Z, Huang J. Clinical analysis of 12 cases of early-onset B group streptococci sepsis in neonates. Clin Pediatr Emerg Med. 2014;21:161–3.

19. Chen Y, Chen R, Wu Z, Lu G. Clinical analysis of 16 cases of neonatal early-onset group B *Streptococcus* sepsis. J Pediatr Pharm. 2014;2011:13–6.

20. Wei C, Li M. Clinical analysis of neonatal group B streptococcal septicemia of eight cases and literature review. Medical Innovation of China. 2014;11:147–50.

21. Huang H, Yu Z, Yang H, Feng J, Liu X. Clinical analysis of neonatal group B streptococcal sepsis. Clin Pediatr Emerg Med. 2014;21:39–40.

22. Long Y, Lai Y, Li Y. Clinical analysis of 16 cases of neonatal group B streptococcal septicaemia. Clin Pediatr Emerg Med. 2014;21:447–8.

23. Zhu M, Zhu J, Li H, Liu P, Lin Z. [Clinical analysis and follow-up of neonatal purulent meningitis caused by group B *streptococcus.*]. Zhonghua Er Ke Za Zhi. 2014;52:133–6. PubMed

24. Liu X, Zhou Q, Shang E, Cheng Y. Analysis of clinical characteristics in neonatal septicemia patients with *Streptococcus agalactiae* and its clinical medication. Anti Infect Pharm. 2015;12:835–7.

25. Zhang S, Luo X, Zhou T, Fu S, Zhu J. Clinical features and therapeutic strategies of late-onset *Streptococcus agalactiae* meningitis. Chin Gen Pract. 2015;18:3633–5.

26. Zeng S, Zhao W, Wang H, Qiu H, Tang X, Feng Z. Study on the serotype of *Streptococcus agalactiae* in neonatal sepsis. Zhongguo Fuyou Baojian. 2015;30:6028–30.

27. Li K, Zhang Y, Du L, Yue W. Clinical analysis and follow-up of 27 cases of neonatal group B streptococcal septicaemia. Shenzhen J Integr Tradit Chin West Med. 2015;15:103–5.

28. Wang Q, Su W. Clinical analysis of 15 cases of neonatal group B streptococcal septicaemia. Zhejiang JITCWM. 2015;2.

29. Wang Y, Lu W, Zhou L. The study of risk factors for neonatal *Streptococcus agalactiae* infection and sensitivity analysis of antibacterials. Int J Lab Med. 2015;36:1065–7.

30. Luo M, Zhang Y, Weng Z, Ou Q, Xiao X. Clinical features of early-onset neonatal septicaemia caused by group B *Streptococcus.* Guangzhou Med J. 2015;46:36–9.

31. Zhao N, Wang P, Lu W, He J, Gu R, Jiang C. Clinical analysis of neonatal purulent meningitis caused by group B *Streptococcus.* Clin Pediatr Emerg Med. 2015;22:177–9.

32. Lei M, Zhang Y, Guo J. Clinical analysis of purulent meningitis related to *Streptococcus agalactiae* in 22 newborn and infants. Zhongguo Shiyong Erke Zazhi. 2015;30:696–700.

33. Liu H, Zeng H, Wang W, Deng Q, Margarit I, Rinaudo CD, et al. Estimating the burden of invasive Group B Streptococcal disease in young infants in southern mainland China: an observational study. Int J Clin Exp Med. 2015;8:13699–707. PubMed

34. Rivera L, Sáez-Llorens X, Feris-Iglesias J, Ip M, Saha S, Adrian PV, et al. Incidence and serotype distribution of invasive group B streptococcal disease in young infants: a multi-country observational study. BMC Pediatr. 2015;15:1–9. PubMed https://doi.org/10.1186/s12887-015-0460-2

35. Zhang J, Zhao R, Dong Y, Zheng Y. Invasive group B streptococcal infection in infants in Shenzhen, China. Int J Clin Exp Med. 2015;8:2939–43. PubMed

36. Liu Z, Xu J, Wang R, Wu L, Chen D. Clinical analysis of 33 cases of neonatal group B Streptococcal sepsis. Clin Pediatr Emerg Med. 2016;23:248–51.

37. Zhang X, Liu K, Wang C. Wu f, Guan H. Clinical analysis of 11 cases of neonatal group B Streptococcal infection. Chin Remed Clin. 2016;9:1360–2.

38. Li L, Wu W, Wu B, Wang S. The relevance of genotype and clinical manifestations of group B *Streptococcus* invasion infection in neonates. Clin J Neonatol. 2016;31:272–5.

39. Li Y, Du F. Clinical analysis of neonatal GBS infection in the third staff hospital of Baogang group. Journal of Inner Mongolia University for Nationalities. 2016;31:525–6.

40. Yang H, Li J. Clinical and prognostic analysis of sepsis caused by *Streptococcus agalactiae* combined with purulent meningitis in 12 neonates J. Clin Pediatr. 2016;34:181–4.

41. Shen Y, Liu H, Qi Y, Dong S, Jin F, Weng J, et al. Retrospective study of group B haemolytic streptococci sepsis in newborn. J Shanxi Med Univ. 2016;47:1041–5.

42. Cai Y, Lin N, Fang X. Study on 15 cases of neonatal group B *Streptococcus* sepsis. Chin J School Doctor. 2016;30:386–8.

43. Lai J, Zheng Z, Lin X, Zhu Y, Lin Y. Clinical analysis of 36 cases of neonatal early onset B *Streptococcus* infection. J Frontier Med. 2016;6:78–9.

44. Zhao L. Clinical characteristics and medication analysis of neonatal *Streptococcus agalactiae* sepsis. Chin Mod Doctor. 2016;54:56–8.

45. Ju H, Bei F, Sun J. Clinical analyses of 16 neonatal group B *Streptococcus* meningitis cases. Clin J Neonatol. 2016;31:178–81.

46. Huang L, Liu H, Huang Y, Guan X, Zhong H, Xie Y, et al. Drug sensitivity analysis of neonatal sepsis and meningitis group B *Streptococcus* isolates in Guangzhou. Guangdong Med J. 2016;37:1873–6.

47. Yue D. Analysis of infection status of neonatal β-hemolytic streptococcus. Chin J Clinical Rational Drug Use. 2017;10:151–2.

48. Qiao L, Ma S, Li D, Yu H, Sun Y. Analysis of 24 cases of infants with late-onset group B streptococci purulent meningitis. Shandong Yiyao. 2017;57:80–2.

49. Guan X, Mu X, Huang Y, Zhong H, Deng Q, Liu H. Epidemiological characteristics of invasive group B streptococcal disease of young infants. Guangzhou Med J. 2017;48:11–4.

50. Liu W, Li H. Clinical analysis of 15 cases of neonatal group B streptococcal septicaemia. Chin J Woman Child Health Research. 2017;28:600–2.

51. Lv C. Clinical Observation on Neonatal *Streptococcus agalactiae* Septicemia complicated with meningitis. China Continuing Medical Education. 2017;9:71–2.

52. Zhou Y, Wang L, Fang Y. Research on the serum type and drug resistance of newborn bloodstream infection caused by group B *Streptococcus.* Chin J Health Lab Tec. 2017;27:1190–3.

53. Zhang J, Deng J, Dong Y, Zhang L, Zhang R, Jia C. Clinical analysis of 55 infants with group B *Streptococcus* bloodstream infection. Chin J Infect Dis. 2017;35:214–7.

54. Zhang N, Yang N, Qu N, Li Z. Clinical analysis of purulent meningitis caused by *Streptococcus agalactiae* in young infants. Zhonghua Shiyong Erke Linchuang Zazhi. 2017;32:1571–4.

55. Wang Y, Li J, Ye S, Li H, Li W. Clinical characteristics of neonatal meningitis caused by *Streptococcus agalactiae.* Pract Clin Med. 2017;18:76–8.

56. Shenzhen NGIRCGi. The clinical study on the early onset and late onset B group haemolytic *Streptococcus* infection in neonates. Clin J Neonatol. 2017;32:241–4.

57. Zhang Sheng ZL, Qiuping L, Xiujuan W, Jie X, Yupei Z, Zhichuan F. Clinical distribution and antimicrobial resistance of *Streptococcus agalactiae* in neonatal intensive care unit. Can J Infect Control. 2017;16:804–6.

58. Tan K, Lu Y, Zhang N. Clinical analysis of 20 cases of neonatal group B streptococcal septicaemia. Guangdong Yixue. 2017;S1:176–8.

59. Zhao T. Study on drug resistance of neonatal *Streptococcus agalactiae sepsis.* Med J Chinese People's Health. 2017;29:27–8.

60. Ma H, Wang Y, Huang Z, Ran M, Tan S, Huang J. Perinatal clinical features of early-onset neonates with group B streptococcal septicemia. J Frontier Med. 2017;7:177–8.

61. Huang W, Lin G, Liu G, Wei Q. Clinical analysis of 30 cases of neonatal group B streptococcal sepsis. Zhonghua Shiyong Erke Linchuang Zazhi. 2017;32:1721–4.

62. Chen IL, Chiu NC, Chi H, Hsu CH, Chang JH, Huang DT, et al. Changing of bloodstream infections in a medical center neonatal intensive care unit. J Microbiol Immunol Infect. 2017;50:514–20. PubMed https://doi.org/10.1016/j.jmii.2015.08.023

63. Chen H, Hu Y, Zhang H, Yang J, Lin M, Zheng J. Clinical features of neonatal group B *Streptococcus* for septicaemia and its risk factors analysis. Chin J Health Lab Tec. 2018;28:300–2.

64. Guan X, Mu X, Ji W, Yuan C, He P, Zhang L, et al. Epidemiology of invasive group B streptococcal disease in infants from urban area of South China, 2011–2014. BMC Infect Dis. 2018;18:14. PubMed https://doi.org/10.1186/s12879-017-2811-0

65. Reisner DP, Haas MJ, Zingheim RW, Williams MA, Luthy DA. Performance of a group B streptococcal prophylaxis protocol combining high-risk treatment and low-risk screening. Am J Obstet Gynecol. 2000;182:1335–43. PubMed https://doi.org/10.1067/mob.2000.106246

66. Chang Chien HY, Chiu NC, Li WC, Huang FY. Characteristics of neonatal bacterial meningitis in a teaching hospital in Taiwan from 1984-1997. J Microbiol Immunol Infect. 2000;33:100–4. PubMed

67. Zhong Y, Wu M, Tong Y, Shen A, Yang Y. A study of neonatal group B streptococcal infection. Chin J Perinat Med. 2002;5:38–41.

68. Liao CH, Huang LM, Lu CY, Lee CY, Hsueh PR, Tsao PN, et al. Group B *streptococcus* infection in infancy: 21-year experience. Acta Paediatr Taiwan. 2002;43:326–9. PubMed

69. Tiskumara R, Fakharee SH, Liu CQ, Nuntnarumit P, Lui KM, Hammoud M, et al.; Asia-Pacific Neonatal Infections Study. Neonatal infections in Asia. Arch Dis Child Fetal Neonatal Ed. 2009;94:F144–8. PubMed https://doi.org/10.1136/adc.2008.139865

70. Lin MC, Chi H, Chiu NC, Huang FY, Ho CS. Factors for poor prognosis of neonatal bacterial meningitis in a medical center in Northern Taiwan. J Microbiol Immunol Infect. 2012;45:442–7. PubMed https://doi.org/10.1016/j.jmii.2011.12.034

71. Ye F, Chang H. Pathogen distribution and antimicrobial resistance of 43 cases of early onset neonatal septicaemia. Clin J Neonatol. 2013;28:85–7.

72. Zhang J, Li B, Dong Y. 5 cases of infantile *Streptococcus agalactiae* septicaemia. J Chin Pediatr. 2013;31:189–90.

73. Lin Z, Wang J. Clinical analysis of 22 cases of neonatal B group hemolytic streptococcal infection. J Chin Physician. 2013;15:1718–9.

74. Tan J, Zhu X, Zhou Y, Mao L. Clinical treatment exploration of 60 cases of neonatal group B streptpcoccal meningitis. Zhongguo Fuyou Baojian. 2014;29:65–7.

75. Chu SM, Hsu JF, Lee CW, Lien R, Huang HR, Chiang MC, et al. Neurological complications after neonatal bacteremia: the clinical characteristics, risk factors, and outcomes. PLoS One. 2014;9:e105294. PubMed https://doi.org/10.1371/journal.pone.0105294

76. Zhang J, Dong Y, Zhao R, Zheng Y. Infant with Group B streptococcal infection: a retrospective analysis of 35 cases. Zhongguo Shiyong Erke Zazhi. 2015;30:215–8.

77. Li L, Wu W, Wu B, Wang S. The relevance between serotypes and clinical characteristics of neonatal infection due to Group B streptococcus and antibiotic sensitivity of serotypes isolated from these infants. Clin J Neonatol. 2015;30:339–42.

78. Mu L, Kuang L, Zhou W, Su M, Jiang Y. Clinical characteristics and antimicrobial resistance analysis of neonatal streptococci infection. Guizhou Med J. 2015;39:644–5.

79. Zhong H, Guan X, Xie Y, Huang L, Wu X. Distribution of serotypes and drug sensitivity analysis of group B *Streptococcus* among infants in Guangzhou area. Zhongguo Fuyou Baojian. 2015;30:6261–3.

80. Zhong H, Guan X, Xie Y, Huang L, Huang Y, Liu H. Infection distribution and drug sensitivity analysis of *Streptococcus agalactiae* in infants and young children. Int J Lab Med. 2015;36:2907–9.

81. Wang P, Ma Z, Tong J, Zhao R, Shi W, Yu S, et al. Serotype distribution, antimicrobial resistance, and molecular characterization of invasive group B *Streptococcus* isolates recovered from Chinese neonates. Int J Infect Dis. 2015;37:115–8. PubMed https://doi.org/10.1016/j.ijid.2015.06.019

82. Li L, Wu W, Wu B, Wang S. The relevance of genotypes and clinical manifestations of group B streptococcus invasion infection in neonates. Clin J Neonatol. 2016;31:272–5.

83. Wang Y. Clinical features of early onset and late onset sepsis in neonates. Chin J Mod Drug Appl. 2016;6.

84. Geng H, Yang B, Zhu X. Clinical analysis of 53 cases of neonatal meningitis and the characteristics of *Streptococcus agalactiae* meningitis. Zhonghua Linchuang Yishi Zazhi. 2016;10:751–4.

85. Huang J. Analysis on the antimicrobial resistance of *Streptococcus agalactiae* in Department of Neonatology. Chin J Clinical Rational Drug Use. 2016;9:21–2.

86. Hua CZ, Yu H, Zhuang JQ, Li XL, Xu HM, Luo QE, et al. An analysis of 181 cases with blood stream infection caused by *Streptococcus agalactiae* in children from 2011 to 2015: a multi-center retrospective study. Zhonghua Er Ke Za Zhi. 2016;54:577–81. PubMed

87. Ding Y, Chen Z, Li R, Lu Y. 10 cases of clinical analysis on group B haemolytic *Streptococcus* meningitis of neonates. Chin Med Pharm. 2017;7:254–6.

88. Wang Y, Chen J, Wei B, Jiang Y, Fu J. Epidemiological survey of neonatal group B hemolytic *Streptococcus.* Zhongguo Fuyou Baojian. 2017;32:2440–2.

89. Jing L, Li Y, Meng D, Wei Q. Multivariate regression analysis of risk factors for neonatal early-onset group B hemolytic streptococcus infection. Chin Mod Doctor. 2017;55:49–51.

90. Wu IH, Tsai MH, Lai MY, Hsu LF, Chiang MC, Lien R, et al. Incidence, clinical features, and implications on outcomes of neonatal late-onset sepsis with concurrent infectious focus. BMC Infect Dis. 2017;17:465. PubMed https://doi.org/10.1186/s12879-017-2574-7

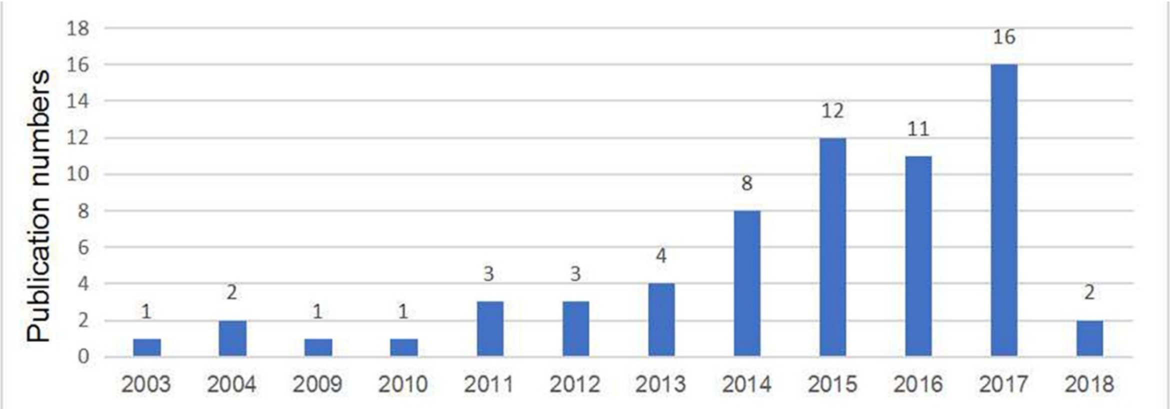

Appendix Figure 1. Publication year of included studies of infants invasive group B *Streptococcus* disease (n = 64) In 2018, we only searched articles published before March16, 2018.

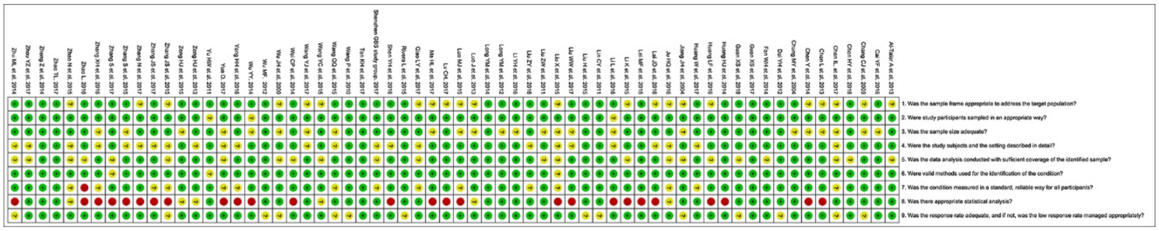

Appendix Figure 2. Risk for bias in the studies. Colored circles indicate different risks. Green, low risk; yellow, unknown risk; red, high risk.

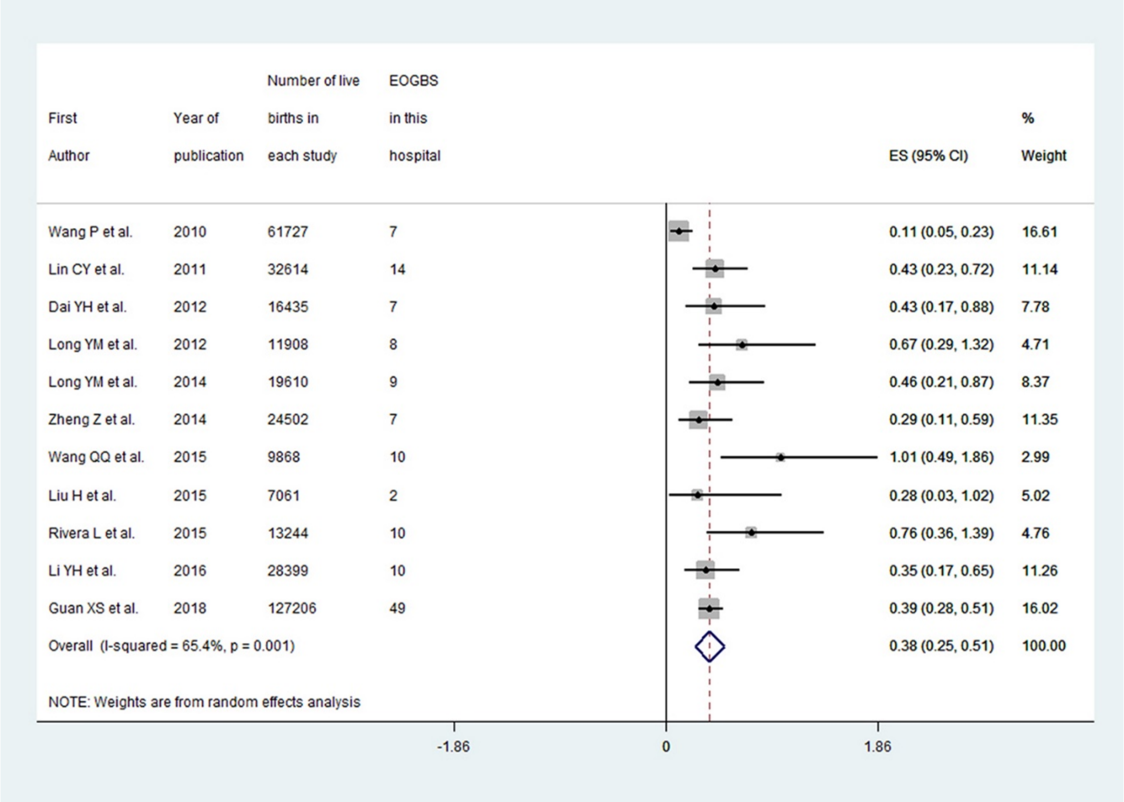

Appendix Figure 3. Incidence risk for early-onset group B *Streptococcus* (EOGBS) disease (n = 11). Vertical dashed line indicates a visual assessment of heterogeneity of the studies. If the vertical line can be drawn, forest plots indicate that all studies are similar enough to be included for meta-analysis. Error bars indicate 95% CI. ES, effect size; GBS, group B *Streptococcus* disease.

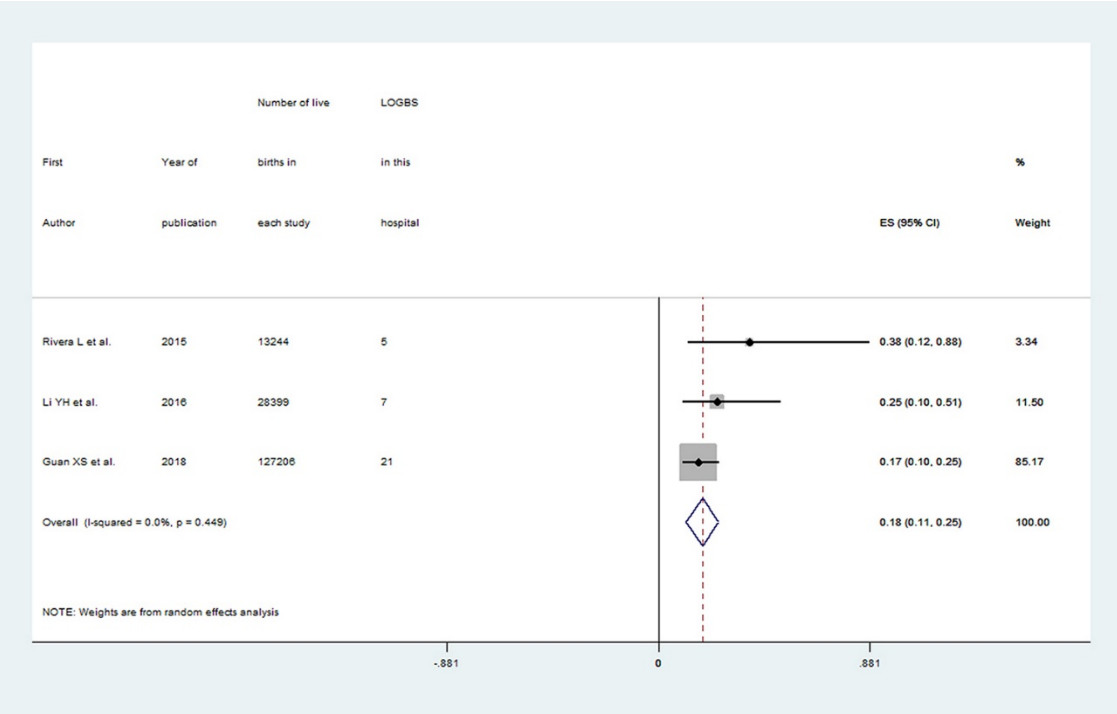

Appendix Figure 4. Incidence risk for late-onset **(**age 7–89 days) group B *Streptococcus* (LOGBS) disease (n = 3). Vertical dashed line indicates a visual assessment of heterogeneity of the studies. If the vertical line can be drawn, forest plots indicate that all studies are similar enough to be included for meta-analysis. Shaded areas indicate relative weight that each individual study contributes to the overall pooled effect. Error bars indicate 95% CI. ES, effect size.

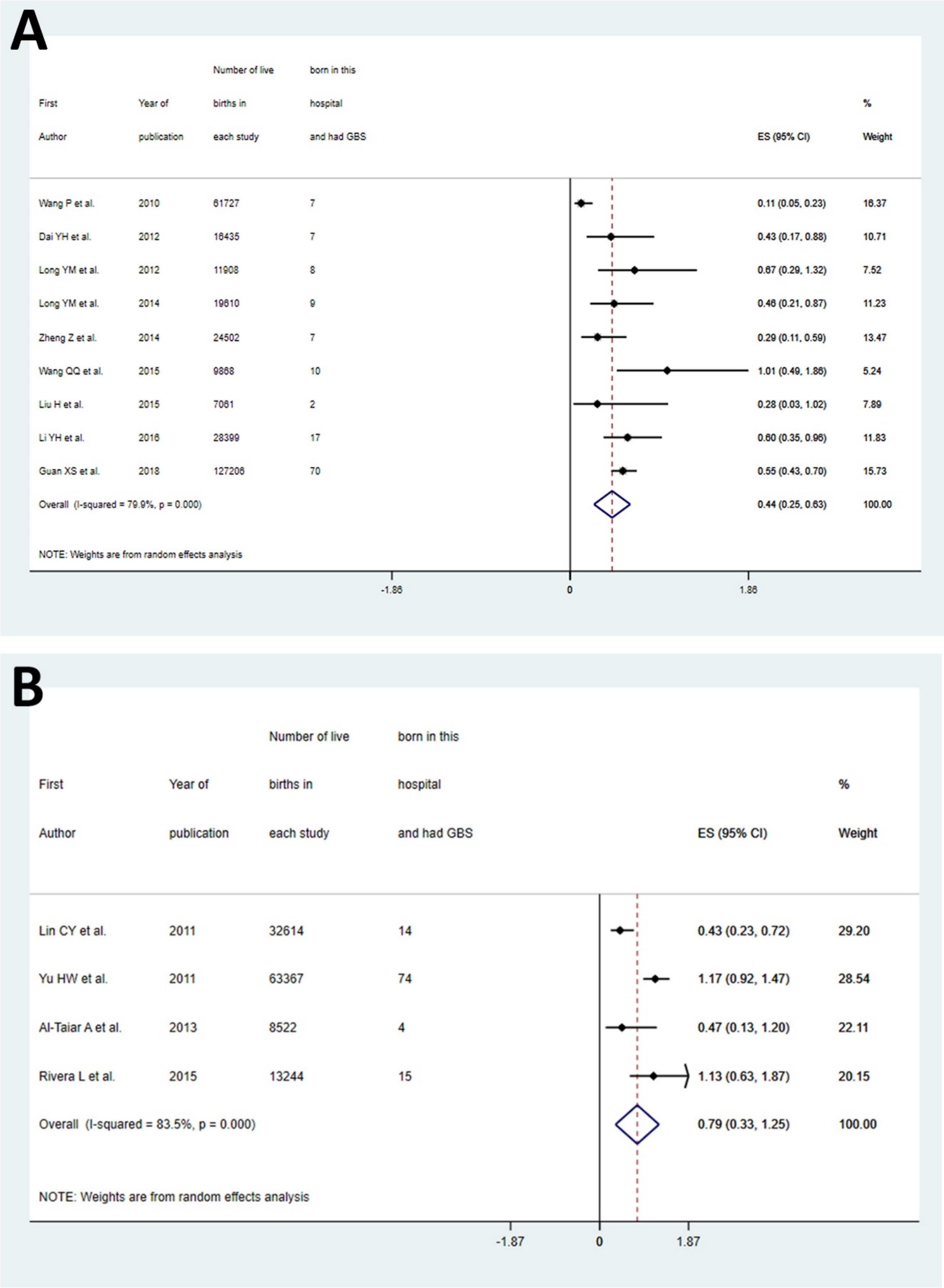
A
First Author
Year of publication
Number of live births in each study
born in this hospital and had GBS
ES (95% CI)
% Weight
Wang P et al. 2010 61727 7 0.11 (0.05, 0.23) 16.37
Dai YH et al. 2012 16435 7 0.43 (0.17, 0.88) 10.71
Long YM et al. 2012 11908 8 0.67 (0.29, 1.32) 7.52
Long YM et al. 2014 19610 9 0.46 (0.21, 0.87) 11.23
Zheng Z et al. 2014 24502 7 0.29 (0.11, 0.59) 13.47
Wang QQ et al. 2015 9888 10 1.01 (0.49, 1.86) 5.24
Liu H et al. 2015 7061 2 0.28 (0.03, 1.02) 7.89
Li YH et al. 2016 28399 17 0.60 (0.35, 0.96) 11.83
Guan XS et al. 2018 127206 70 0.55 (0.43, 0.70) 15.73
Overall (I-squared = 79.9%, p = 0.000) 0.44 (0.25, 0.63) 100.00
NOTE: Weights are from random effects analysis
-1.86
0
1.86
B
First Author
Year of publication
Number of live births in each study
born in this hospital and had GBS
ES (95% CI)
% Weight
Lin CY et al. 2011 32614 14 0.43 (0.23, 0.72) 29.20
Yu HW et al. 2011 63367 74 1.17 (0.92, 1.47) 28.54
Al-Taiar A et al. 2013 8522 4 0.47 (0.13, 1.20) 22.11
Rivera L et al. 2015 13244 15 1.13 (0.63, 1.87) 20.15
Overall (I-squared = 83.5%, p = 0.000) 0.79 (0.33, 1.25) 100.00
NOTE: Weights are from random effects analysis
-1.87
0
1.87

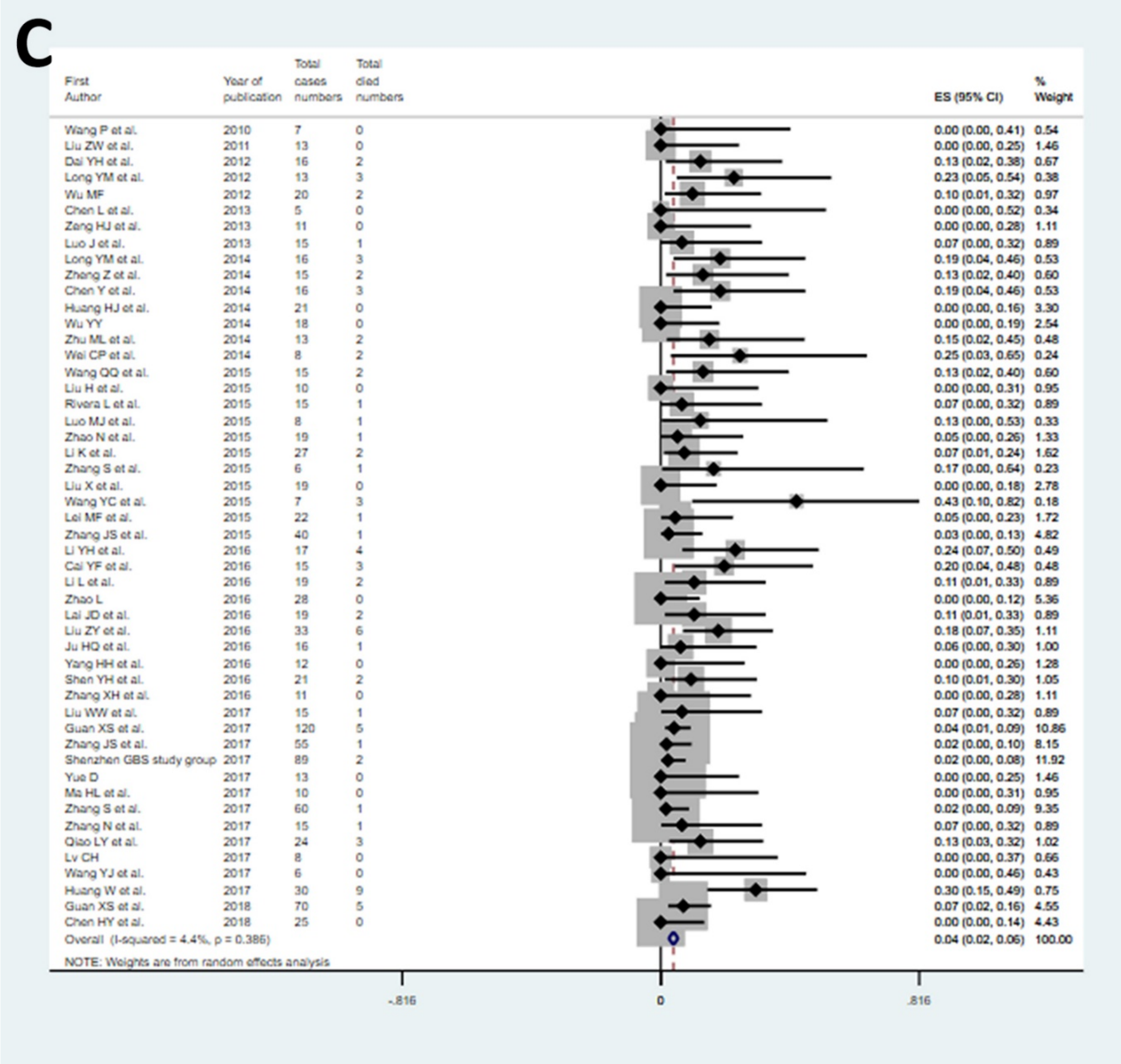
C
First Author
Year of publication
Total cases numbers
Total died numbers
ES (95% CI)
% Weight
Wang P et al. 2010 7 0 0.00 (0.00, 0.41) 0.54
Liu ZW et al. 2011 13 0 0.00 (0.00, 0.25) 1.46
Dai YH et al. 2012 16 2 0.13 (0.02, 0.38) 0.67
Long YM et al. 2012 13 3 0.23 (0.05, 0.54) 0.38
Wu MF 2012 20 2 0.10 (0.01, 0.32) 0.97
Chen L et al. 2013 5 0 0.00 (0.00, 0.52) 0.34
Zeng HJ et al. 2013 11 0 0.00 (0.00, 0.28) 1.11
Luo J et al. 2013 15 1 0.07 (0.00, 0.32) 0.89
Long YM et al. 2014 16 3 0.19 (0.04, 0.46) 0.53
Zhang Z et al. 2014 15 2 0.13 (0.02, 0.40) 0.60
Chen Y et al. 2014 16 3 0.19 (0.04, 0.46) 0.53
Huang HJ et al. 2014 21 0 0.00 (0.00, 0.16) 3.30
Wu YY 2014 18 0 0.00 (0.00, 0.19) 2.54
Zhu ML et al. 2014 13 2 0.15 (0.02, 0.45) 0.48
Wei CP et al. 2014 8 2 0.25 (0.03, 0.65) 0.24
Wang QQ et al. 2015 15 2 0.13 (0.02, 0.40) 0.60
Liu H et al. 2015 10 0 0.00 (0.00, 0.31) 0.95
Rivera L et al. 2015 15 1 0.07 (0.00, 0.32) 0.89
Luo MJ et al. 2015 8 1 0.13 (0.00, 0.53) 0.33
Zhao N et al. 2015 19 1 0.05 (0.00, 0.26) 1.33
Li K et al. 2015 27 2 0.07 (0.01, 0.24) 1.62
Zhang S et al. 2015 6 1 0.17 (0.00, 0.64) 0.23
Liu X et al. 2015 19 0 0.00 (0.00, 0.18) 2.78
Wang YC et al. 2015 7 3 0.43 (0.10, 0.82) 0.18
Lei MF et al. 2015 22 1 0.05 (0.00, 0.23) 1.72
Zhang JS et al. 2015 40 1 0.03 (0.00, 0.13) 4.82
Li YH et al. 2016 17 4 0.24 (0.07, 0.50) 0.49
Cai YF et al. 2016 15 3 0.20 (0.04, 0.48) 0.48
Li L et al. 2016 19 2 0.11 (0.01, 0.33) 0.89
Zhao L 2016 28 0 0.00 (0.00, 0.12) 5.36
Lai JD et al. 2016 19 2 0.11 (0.01, 0.33) 0.89
Liu ZY et al. 2016 33 6 0.18 (0.07, 0.35) 1.11
Ju HQ et al. 2016 16 1 0.06 (0.00, 0.30) 1.00
Yang HH et al. 2016 12 0 0.00 (0.00, 0.26) 1.28
Shen YH et al. 2016 21 2 0.10 (0.01, 0.30) 1.05
Zhang XH et al. 2016 11 0 0.00 (0.00, 0.28) 1.11
Liu WW et al. 2017 15 1 0.07 (0.00, 0.32) 0.89
Guan XS et al. 2017 120 5 0.04 (0.01, 0.09) 10.86
Zhang JS et al. 2017 55 1 0.02 (0.00, 0.10) 8.15
Shenzhen GBS study group 2017 89 2 0.02 (0.00, 0.08) 11.92
Yue D 2017 13 0 0.00 (0.00, 0.25) 1.46
Ma HL et al. 2017 10 0 0.00 (0.00, 0.31) 0.95
Zhang S et al. 2017 60 1 0.02 (0.00, 0.09) 9.35
Zhang N et al. 2017 15 1 0.07 (0.00, 0.32) 0.89
Qiao LY et al. 2017 24 3 0.13 (0.03, 0.32) 1.02
Lv CH 2017 8 0 0.00 (0.00, 0.37) 0.66
Wang YJ et al. 2017 6 0 0.00 (0.00, 0.46) 0.43
Huang W et al. 2017 30 9 0.30 (0.15, 0.49) 0.75
Guan XS et al. 2018 70 5 0.07 (0.02, 0.16) 4.55
Chen HY et al. 2018 25 0 0.00 (0.00, 0.14) 4.43
Overall (I-squared = 4.4%, p = 0.386) 0.04 (0.02, 0.06) 100.00
NOTE: Weights are from random effects analysis
-.816
0
.816

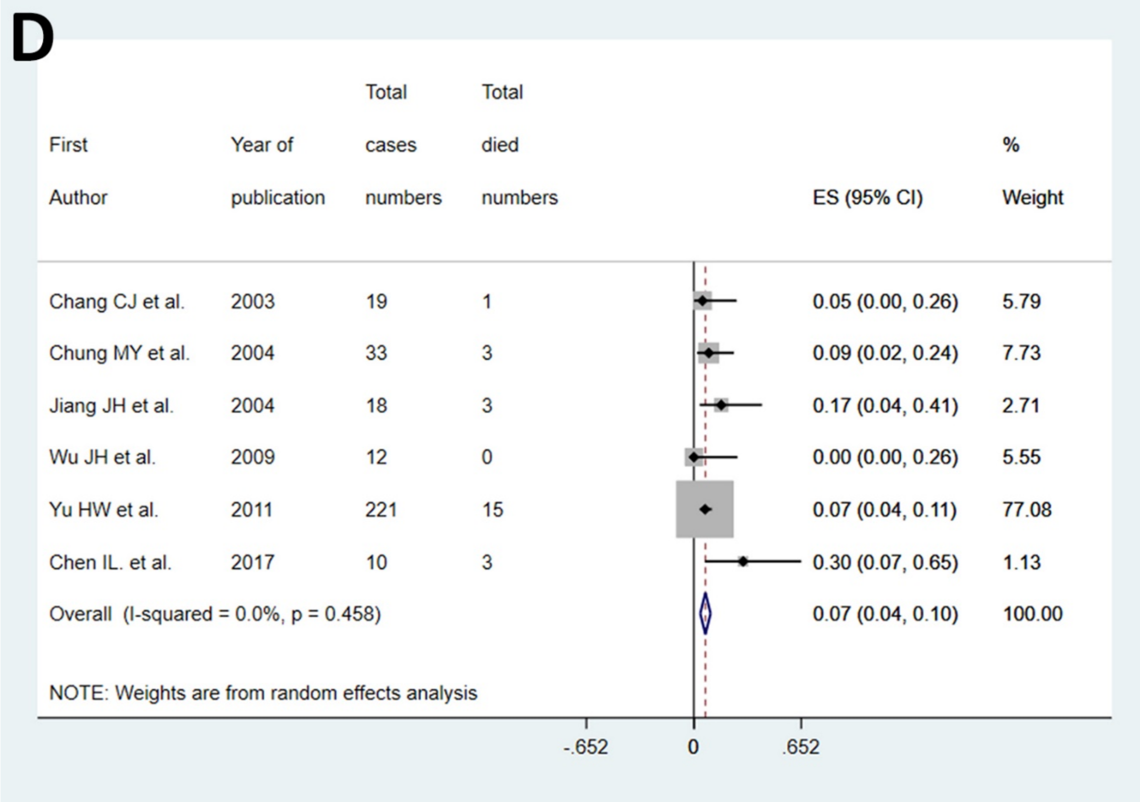

Appendix Figure 5. Sensitivity analysis of GBS invasive diseases incidence studies. A) Total incidence of GBS invasive disease in Mainland China; B) total incidence of GBS invasive disease in Taiwan, Hong Kong, and Macau; C) total CFR of GBS invasive disease in Mainland China; D) total CFR of GBS invasive disease in Taiwan, Hong Kong, and Macau. Vertical dashed line indicates a visual assessment of heterogeneity of the studies. If the vertical line can be drawn, forest plots indicate that all studies are similar enough to be included for meta-analysis. Shaded areas indicate relative weight that each individual study contributes to the overall pooled effect. ES, effect size; GBS, group B *Streptococcus* disease.

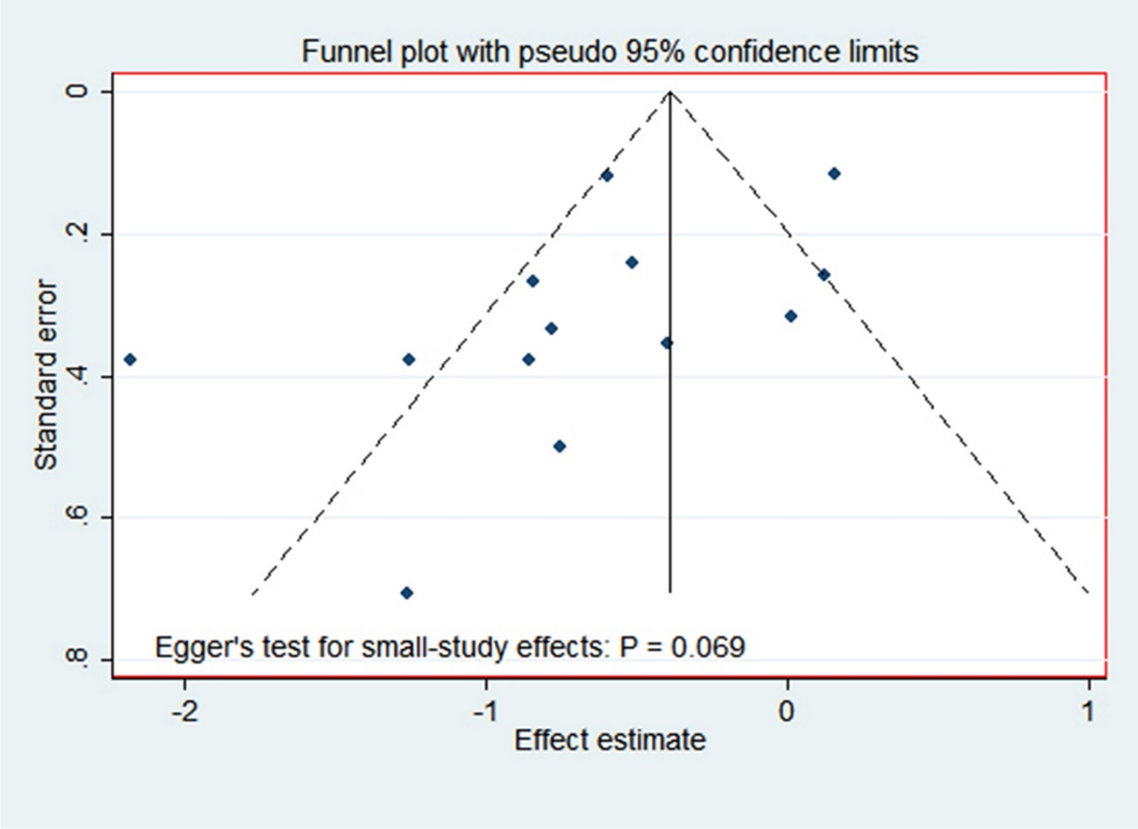

Appendix Figure 6. Funnel plot showing publication bias for group B *Streptococcus* disease.

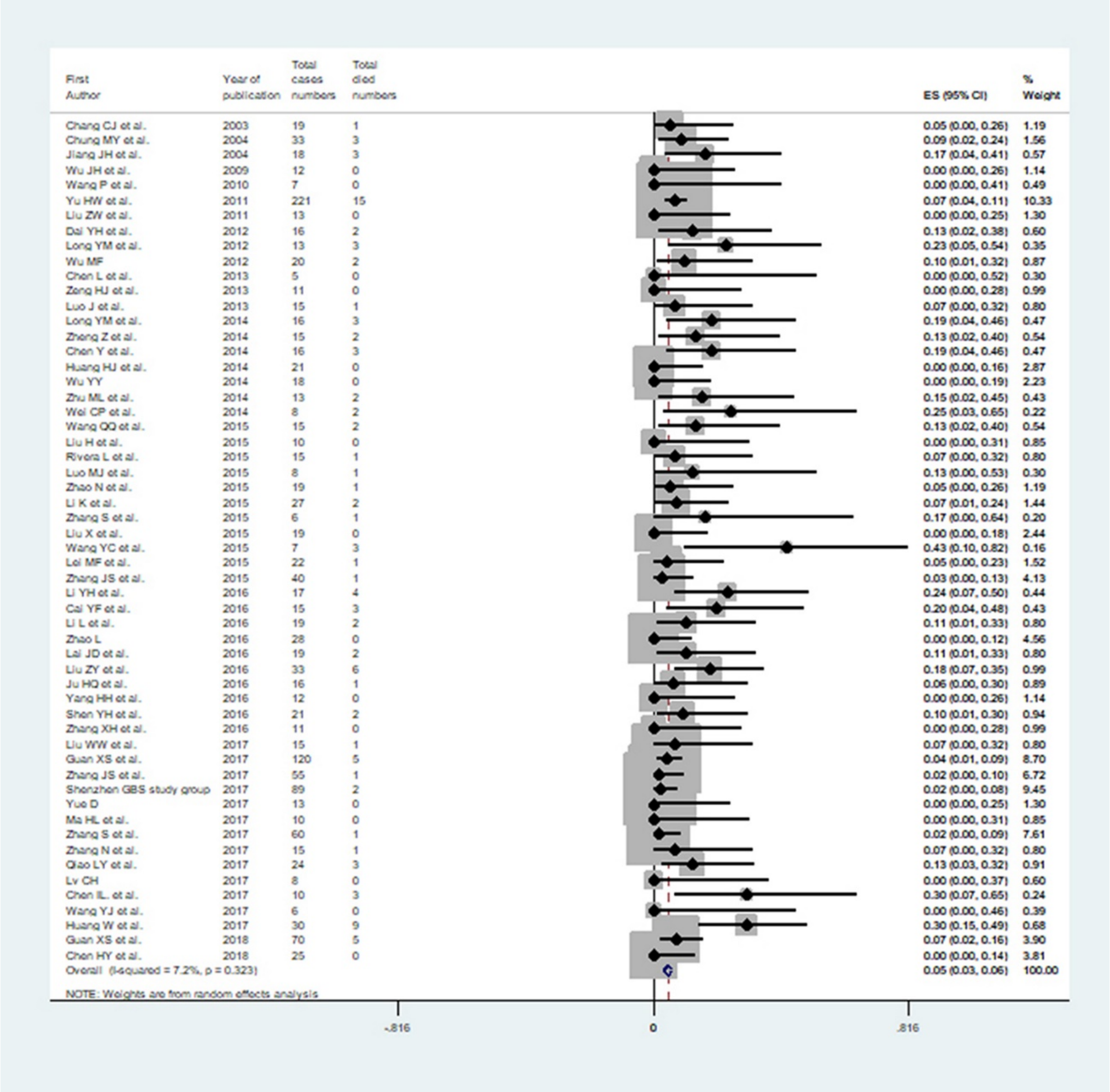

Appendix Figure 7. Case-fatality rate of group B *Streptococcus* (GBS) disease in infants <1–89 days of age (n = 56). Vertical dashed line indicates a visual assessment of heterogeneity of the studies. If the vertical line can be drawn, forest plots indicate that all studies are similar enough to be included for meta-analysis. Shaded areas indicate relative weight that each individual study contributes to the overall pooled effect. CFR, case-fatality rate; ES, effect size; GBS, group B *Streptococcus* disease.

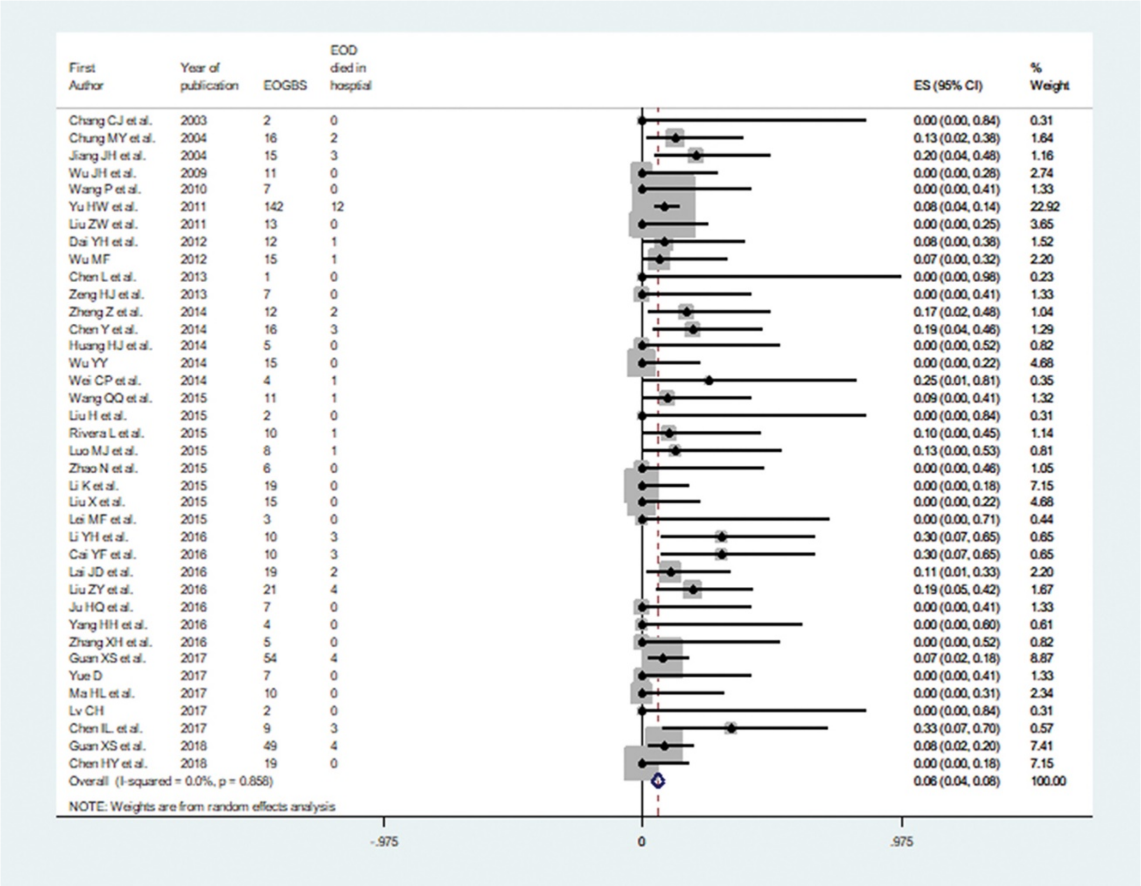

Appendix Figure 8. Case-fatality rate (CFR) of early-onset group B *Streptococcus* (EOGBS) disease (n = 38). Vertical dashed line indicates a visual assessment of heterogeneity of the studies. If the vertical line can be drawn, forest plots indicate that all studies are similar enough to be included for meta-analysis. Shaded areas indicate relative weight that each individual study contributes to the overall pooled effect. EOD, patient died in the hospital; ES, effect size.

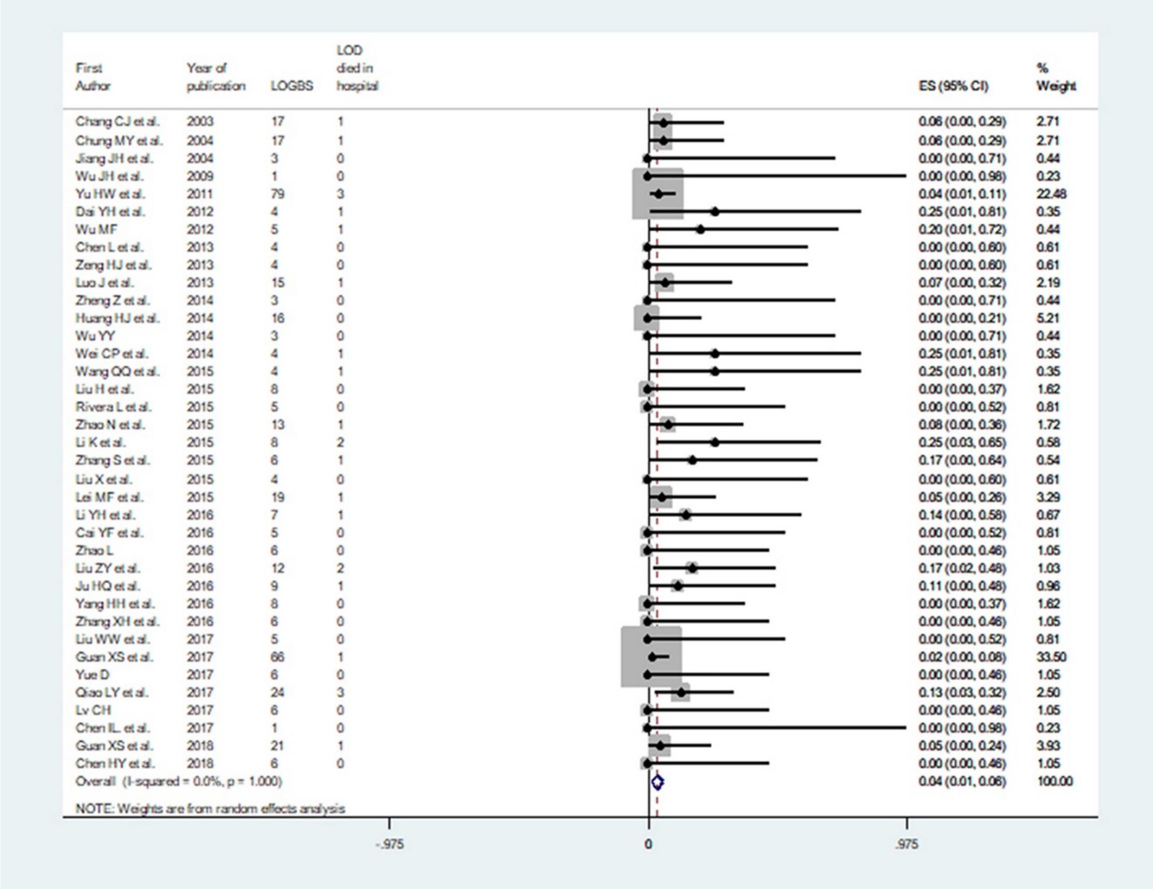

Appendix Figure 9. Case fatality rate (CFR) of late-onset group B *Streptococcus* (LOGBS) disease in children 7–89 days of age (n = 37). Vertical dashed line indicates a visual assessment of heterogeneity of the studies. If the vertical line can be drawn, forest plots indicate that all studies are similar enough to be included for meta-analysis. Shaded areas indicate relative weight that each individual study contributes to the overall pooled effect. LOD, patient died in the hospital; ES, effect size.

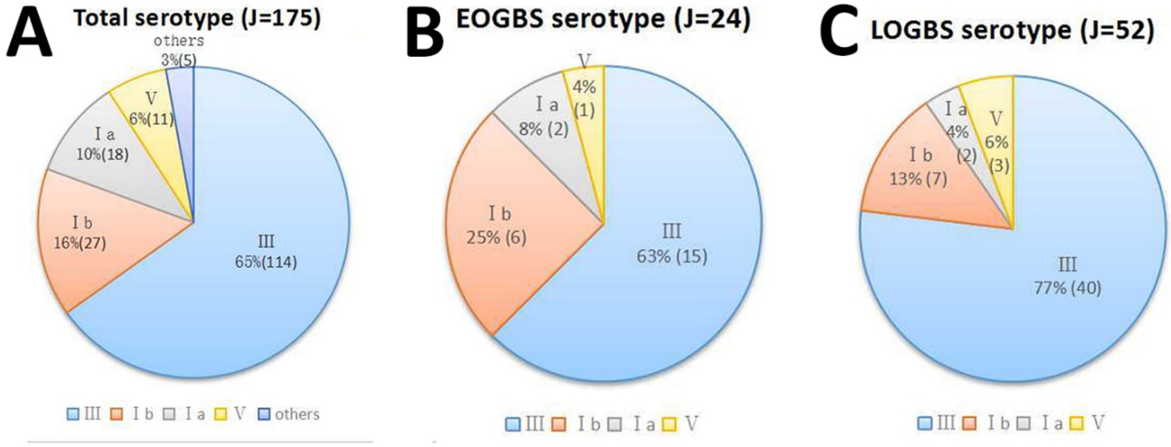

Appendix Figure 10. Serotype distribution of group B *Streptococcus* (GBS) in infants <1–89 days of age with invasive disease. A) Overall serotype distribution of GBS; B) distribution of early-onset GBS disease; C) distribution of late-onset GBS disease. EOGBS, early-onset GBS disease; LOGBS, late-onset GBS disease.
